# Autism-like behaviours and enhanced memory formation and synaptic plasticity in Lrfn2/SALM1-deficient mice

**DOI:** 10.1038/ncomms15800

**Published:** 2017-06-12

**Authors:** Naoko Morimura, Hiroki Yasuda, Kazuhiko Yamaguchi, Kei-ichi Katayama, Minoru Hatayama, Naoko H. Tomioka, Maya Odagawa, Akiko Kamiya, Yoshimi Iwayama, Motoko Maekawa, Kazuhiko Nakamura, Hideo Matsuzaki, Masatsugu Tsujii, Kazuyuki Yamada, Takeo Yoshikawa, Jun Aruga

**Affiliations:** 1 Laboratory for Behavioral and Developmental Disorders, RIKEN Brain Science Institute (BSI) Wako, Saitama 351-0198, Japan; 2 Department of Integrative Physiology, Shiga University of Medical Science, Otsu, Shiga 520-2192, Japan; 3 Education and Research Support Center, Gunma University Graduate School of Medicine, Maebashi, Gunma 371-8511, Japan; 4 Laboratory for Behavioral Genetics, RIKEN Brain Science Institute (BSI) Wako, Saitama 351-0198, Japan; 5 Department of Medical Pharmacology, Nagasaki University Graduate School of Biomedical Sciences, Nagasaki, Nagasaki 852-8523, Japan; 6 Laboratory for Molecular Psychiatry, RIKEN Brain Science Institute (BSI) Wako, Saitama 351-0198, Japan; 7 Department of Neuropsychiatry, Hirosaki University School of Medicine, Hirosaki, Aomori 036-8562, Japan; 8 Research Center for Child Mental Development, University of Fukui, Yoshida-gun, Fukui 910-1193, Japan; 9 Faculty of Contemporary Sociology, Chukyo University, Toyota, Aichi 470-0393, Japan; 10 Support Unit for Animal Experiments, RIKEN Brain Science Institute (BSI) Wako, Saitama 351-0198, Japan

## Abstract

Lrfn2/SALM1 is a PSD-95-interacting synapse adhesion molecule, and human *LRFN2* is associated with learning disabilities. However its role in higher brain function and underlying mechanisms remain unknown. Here, we show that *Lrfn2* knockout mice exhibit autism-like behavioural abnormalities, including social withdrawal, decreased vocal communications, increased stereotyped activities and prepulse inhibition deficits, together with enhanced learning and memory. In the hippocampus, the levels of synaptic PSD-95 and GluA1 are decreased. The synapses are structurally and functionally immature with spindle shaped spines, smaller postsynaptic densities, reduced AMPA/NMDA ratio, and enhanced LTP. *In vitro* experiments reveal that synaptic surface expression of AMPAR depends on the direct interaction between Lrfn2 and PSD-95. Furthermore, we detect functionally defective *LRFN2* missense mutations in autism and schizophrenia patients. Together, these findings indicate that Lrfn2/LRFN2 serve as core components of excitatory synapse maturation and maintenance, and their dysfunction causes immature/silent synapses with pathophysiological state.

## Abstract (web summary)

Lrfn2/SALM1 is a synaptic adhesion molecule, and is known to interact with PSD-95. Here the authors show that Lrfn2 regulates excitatory synapse maturation and maintenance, and that Lrfn2 knockout mice exhibit autism-like behaviours as well as enhanced learning and memory.

Synapse dysfunction is associated with the pathophysiology of various neurodevelopmental disorders such as autism, intellectual disability and schizophrenia^1^,^2^. Genetic studies suggest that the components of excitatory synapses are involved in the pathogenesis^2^,^3^. Mutations in *NRXN1*, *NLGN3/4* and *SHANK1/2/3* alter synaptic function and lead to autism spectrum disorders (ASD), intellectual disability or schizophrenia^2^,^4^,^5^,^6^,^7^. Neurexins (NRXN) and Neuroligins (NLGN) are presynaptic and postsynaptic synaptic adhesion proteins, respectively^6^. Shanks are scaffold proteins in the postsynaptic density (PSD)^4^.

NLGN and Shanks are linked by PSD-95, an abundant PDZ domain–containing scaffold protein^8^,^9^. PSD-95 is a critical postsynaptic regulator that facilitates the formation of the PSD and has multiple protein–protein interaction domains to organize a molecular network linking extracellular to intracellular components. The PDZ domains of PSD-95 associate with the PDZ-binding motif located at the carboxy-termini of transmembrane molecules such as the glutamate receptors^10^,^11^, NLGN and other adhesion molecules^8^,^9^. Recruitment of PSD-95 and other PSD-95-interacting membrane molecules to the synapse has been proposed to lead to trafficking and clustering of AMPA receptors (AMPAR) and NMDA receptors (NMDAR), consequently affecting synaptic morphology, function and plasticity^12^,^13^. Mice with genetic deletion of PSD-95 show enhanced long-term potentiation (LTP) and more silent synapse population by the dampened AMPA function, as well as longer spines and impaired learning^14^,^15^.

Although many postsynaptic adhesion molecules can physically interact with PSD-95 *in vitro*, the major regulator of PSD-95 *in vivo* is unclear. For example, Neuroligin1/2/3 triple-knockout (KO) mice show normal synaptogenesis and synaptic morphology of hippocampal and neocortical neurons^16^. Furthermore, to our knowledge, none of the other PDZ-binding postsynaptic synaptic adhesion proteins, including Neuroligin4 (ref. 17), Lrrtm2 (ref. 18), NGL3 (ref. 19) and SynCAM1 (ref. 20), have been shown to alter the amount or distribution of PSD-95 *in vivo*. It is essential to clarify the postsynaptic protein that regulates PSD-95 in physiological contexts, given its importance in AMPAR regulation.

Lrfn2 (also known as SALM1) is a neuronal single-pass transmembrane protein that physically interacts with PSD-95 through a C-terminal PDZ-binding motif^21^,^22^,^23^. In human, *LRFN2* is associated with autism^24^, learning disabilities^25^, and antisocial personality disorder^26^. Lrfn2 overexpression promotes neurite outgrowth^27^, recruits NMDAR, and enhances the surface expression of transfected GluN2A (NR2A, a glutamate receptor subunit)^23^. Lrfn2 can bind the NMDA receptor GluN1 (NR1) subunit through their extracellular or transmembrane domains^23^. Although previous studies addressed the molecular function of Lrfn2, its physiological role at the synapse is little understood, presumably because no loss-of-function analysis has been performed *in vivo*.

Here, we show that Lrfn2 is a major regulator of PSD-95 in hippocampal neurons and is required for excitatory synapse maturation. *Lrfn2* KO mice showed ASD-like behaviours with enhanced cognitive function. We also carried out resequencing analysis of *LRFN2* in autism and schizophrenia patients and identified functionally impaired missense mutations. These results suggest that Lrfn2 is an integral component of the excitatory postsynaptic assembly, and defects in it could lead to developmental psychiatric illnesses.

## Results

### Characterization and expression pattern of Lrfn proteins

**Figure 1:**
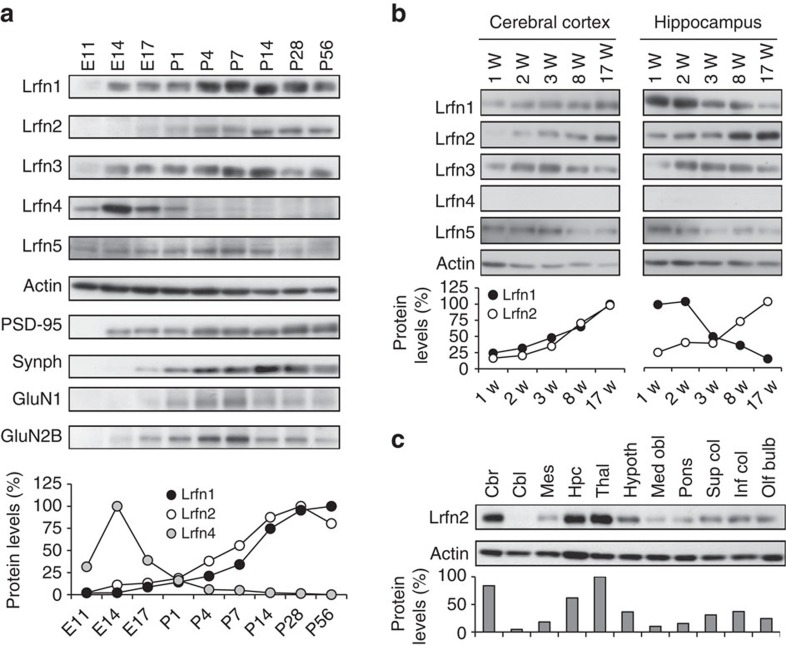
Expression profiles of Lrfn proteins and characterization of Lrfn2 in the mouse brain. (**a**) Developmental expression patterns in mouse brain. Mouse brain extracts (10 μg of protein per lane) at the indicated ages (E, embryonic day; P, postnatal day) were analysed by immunoblotting with the indicated antibodies. Actin was monitored as a loading control. Synph represents synaptophysin. (**b**) Expression profiles of Lrfn proteins in the cerebral cortex and hippocampus during mouse postnatal development (W, postnatal week). Mouse tissue homogenates (5 μg of protein per lane) were analysed by immunoblotting. Actin was monitored as a loading control. Lrfn4 was not detected in the conditions we used. (**a**,**b**) Graphs indicate actin-normalized protein levels of PSD-95-binding Lrfn proteins, based on densitometric quantification of the indicated images. Maximal expression levels are adjusted to 100%. (**c**) Tissue distribution of Lrfn2 protein in 8-week-old mouse brain (10 μg of protein per lane). Graph indicates actin-normalized Lrfn2 protein level. Maximal expression level is adjusted to 100%. (**a**–**c**) Similar results were obtained in 2–6 independent blots except synaptophysin, GluN1, and GluN2B.

A previous study detected *Lrfn* mRNAs in pyramidal neurons in the cerebral cortex and hippocampus of mouse brain^22^. In the present work, we assessed the developmental expression profiles of Lrfn proteins using subtype-specific antibodies (Supplementary Fig. 1a). In whole-brain lysates (Fig. 1a), Lrfn1/SALM2 and Lrfn3/SALM4 were detected from embryonic day 14 to 56 and peaked between postnatal day 4 and 14. Lrfn2/SALM1 and Lrfn5/SALM5 proteins increased in the first postnatal week. Lrfn5 levels decreased after postnatal day 28, whereas Lrfn2 levels were maintained. The majority of Lrfn4/SALM3 expression was observed during embryonic stages, and it became undetectable after birth. Lrfn2 expression increased in the postnatal cerebral cortex and hippocampus, (Fig. 1b), and Lrfn2 was the only Lrfn family member that prominently increased in postnatal hippocampus.

In adulthood, Lrfn2 protein was abundant in the cerebral cortex and hippocampus (Fig. 1c). Mouse Lrfn2 protein was detected in the synaptosomal plasma membrane and PSD fractions (Supplementary Fig. 1b), similar to the distribution of rat Lrfn2 (ref. 23). Epitope tagged mammalian Lrfn2 proteins localize to both dendrites and axons and can be detected in postsynapse, overlapping with PSD-95 (Supplementary Fig. 1c; ref. 23). In an immunoprecipitation experiment using the mouse brain synaptosomal fraction, Lrfn2 co-precipitated not only with PSD-95 (Supplementary Fig. 1d; ref. 23), but also with the GluA1 and GluA2 subunits of AMPAR and the GluN2A and GluN2B subunits of NMDAR (Supplementary Fig. 1d).

### Lrfn2 KO mice exhibit social withdrawal

To understand the role of Lrfn2, we generated *Lrfn2* KO mice (*Lrfn2*^*−/−*^). In the *Lrfn2* null mutation allele (*Lrfn2*^*−*^), *Lrfn2* exon 2, which encodes most of the extracellular region from the amino-terminus to the middle of the FnIII domain, was replaced with a single loxP sequence (Supplementary Fig. 2a–c), and the absence of Lrfn2 protein was confirmed by immunoblotting (Supplementary Fig. 2d). *Lrfn2* KO was not lethal (*Lrfn2*^*+/+*^, 25.9%; *Lrfn2*^*+/−*^, 46.0%; *Lrfn2*^*−/−*^, 28.1% in 913 3- to 8-week-old progenies from interheterozygote mating; no significant differences from the expected mendelian ratio in *χ*^2^ test), and *Lrfn2* KO mice were fertile and displayed no gross defects in brain anatomy (Supplementary Fig. 2e).

**Figure 2:**
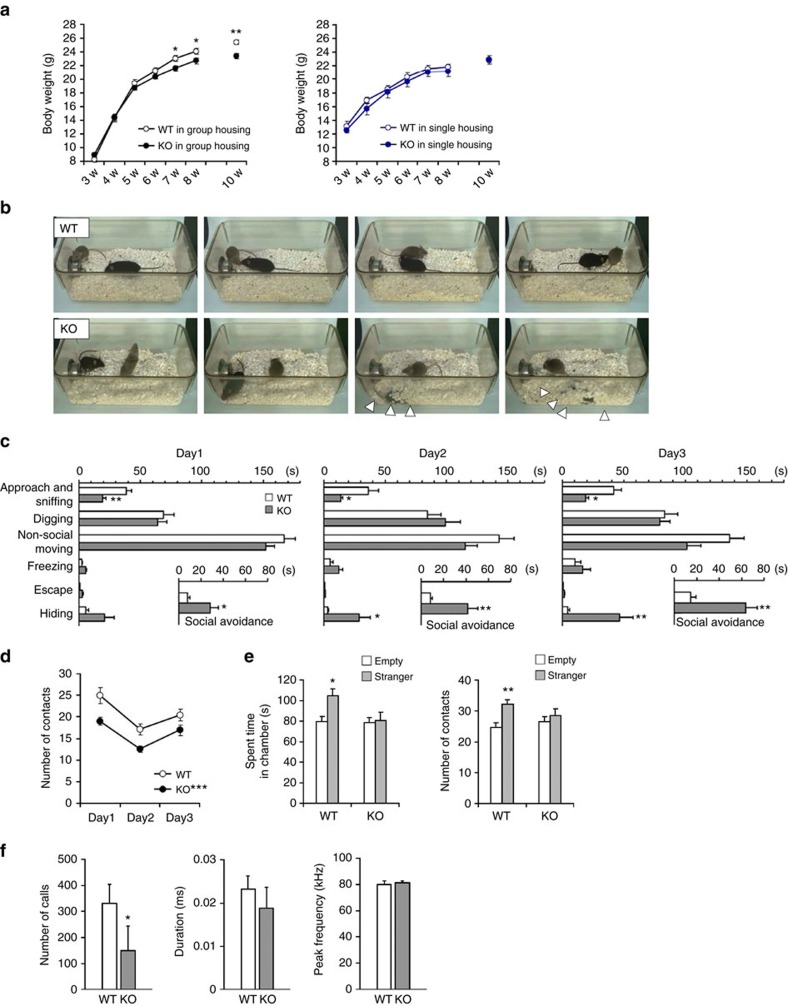
Social withdrawal in *Lrfn2* KO mice. (**a**) Postweaning body weight curve of male WT and *Lrfn2* KO mice in-housed in groups (WT, *n*=10 mice; *Lrfn2* KO, *n*=10 mice from two independent experiments; *P<0.05, **P<0.01, *t*-test) or singly after weaning at 3 weeks (WT, *n*=5 mice; *Lrfn2* KO, *n*=7 mice). (**b**–**d**) Reciprocal social interaction test. The test was conducted on 3 consecutive days, each time with a different intruder mouse (10-week-old DBA2 male) that was new to resident mouse. (**b**) Snapshots of social behaviour in WT and *Lrfn2* KO mice taken from representative experiments (Supplementary Movies 1 and 2). Arrowheads indicate the hiding mouse. (**c**) Time spent in each behaviour during 5 min total observation time. The inset shows the sum of time spent in avoidance behaviour, including escape, freezing and hiding (*n*=10 mice per genotype; *P<0.05, **P<0.01, *t*-test). (**d**) The number of contacts was significantly lower in the KO than in the WT (***P<0.001, two-way ANOVA). (**e**) Social interaction in a 3-chambered approach task. An empty cage or a cage with a stranger mouse (10-week-old DBA2) was placed in each side chamber. *Left*, time spent in each chamber during 5 min total observation time. *Right*, number of contacts with each cage (*n*=16 per genotype; *P<0.05, **P<0.01, one-way ANOVA). (**f**) Courtship vocalizations of adult male mice. USV were recorded from adult males exposed to a female mouse in oestrous. Number, duration, and peak frequency of the USV during the 5-min exposure were quantified (WT, *n*=13 mice; KO, *n*=12 for number; WT, *n*=12; KO, *n*=6 for duration and peak frequency. *P<0.05, *U*-test).

However, *Lrfn2* KO mice showed mild (6–9%) body weight loss in a conventional group housing environment of 4–5 mice per cage (mean body weight±s.e.m. of 8-week-old male wild types (WT), 24.1±0.43 g; male KO, 22.7±0.39 g; female WT, 19.7±0.25 g; female KO, 17.9±0.24 g; *n*=10–19; *P*=0.02 [male] and *P*=0.02 [female] by *t*-test). The body weight difference started 6 weeks postnatally and became obvious at 7–10 weeks (Fig. 2a left). In contrast, when the mice were reared in an isolated housing condition from 3 weeks of age, there were no obvious differences in body weight between WT and *Lrfn2* KO (Fig. 2a right). We speculated that juvenile *Lrfn2* KO mice have altered behavioural responses to sustained social stimuli.

To investigate the social behaviour, we conducted the reciprocal social interaction test (Fig. 2b–d)^28^,^29^. In three trials carried out on 3 consecutive days, *Lrfn2* KO mice showed remarkably reduced social investigation such as approach and sniffing (Fig. 2c), and less physical contact with a DBA2 male mouse (Fig. 2d). Interestingly, *Lrfn2* KO mice frequently hid themselves under the bedding material instead of social investigation (Fig. 2b and Supplementary Movies 1 and 2). This withdrawn behaviour from the social peer was notably enhanced day by day (Fig. 2c). In addition, social avoidance behaviour that includes freezing, escaping and hiding was much more time in *Lrfn2* KO mice than WT mice (insets of Fig. 2c). Together, these observations revealed that *Lrfn2* KO mice show a marked tendency to social withdrawal and avoidance.

We next performed a 3-chambered social approach test as a measure of one-way social activity^30^. WT mice showed normal sociability with a significant preference for a caged stranger mouse (DBA2 male), whereas *Lrfn2* KO mice did not show a significant preference in terms of either time spent in each chamber or number of contacts with the stranger's cage (Fig. 2e).

Having observed the social behaviour abnormalities, we checked the ultrasonic vocalizations (USV) of nursing infants and mating adults. Although there was no significant difference in the properties of isolation-induced USV of nursing infants (Supplementary Fig. 3a), mature *Lrfn2* KO males encountering oestrous females emitted fewer USV than WT males (Fig. 2f). Thus, communication skills are impaired in the *Lrfn2* KO, consistent with the tendency for social withdrawal.

We also checked if some physical or mental changes associate with the *Lrfn2* social phenotype. As a result, there were no abnormalities in tests of olfaction (Supplementary Fig. 3b), locomotion (Supplementary Fig. 3c,e), spontaneous motor activities in home cages (Supplementary Fig. 3d) and motor coordination on an accelerating rotarod (Supplementary Fig. 3f), or in conventional anxiety-related behavioural tasks such as light/dark box test (Supplementary Fig. 3g), elevated plus-maze test (Supplementary Fig. 3h), and open field test (the time spent in the centre area, Supplementary Fig. 3c).

### Lrfn2 KO mice exhibit repetitive behaviours and impaired PPI

**Figure 3:**
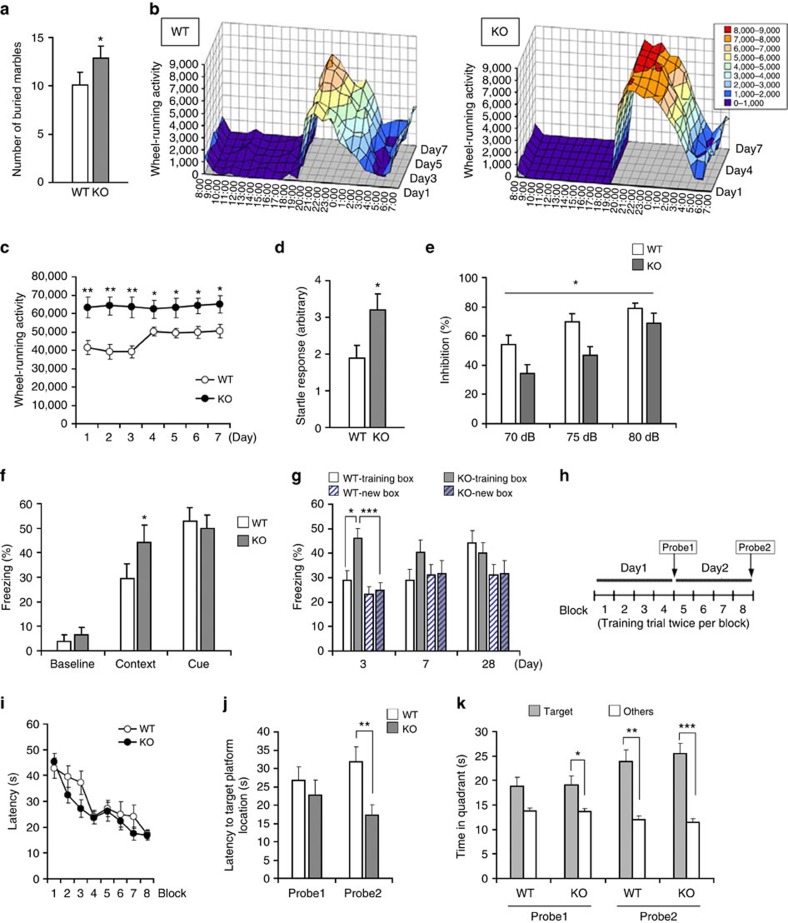
Repetitive behaviours and sensory dysfunction and enhanced memory function of *Lrfn2* KO mice. (**a**) Marble burying test. Mice were exposed to 15 unburied marbles on bedding material for 20 min, and then the buried marbles were counted. *n*=8 per genotype; *P<0.05, *U*-test. (**b**,**c**) Wheel-running test. (**b**) Wheel-running activity. Time bin, 1 h. *Lrfn2* KO mice displayed excessive wheel-running activity during the dark phase. (**c**) Mean of wheel-running activity for each day (*n*=10 per genotype; *P<0.05, **P<0.01, *t*-test). (**d**) Auditory startle response evoked by a 120-dB acoustic stimulus (*n*=10 per genotype; *P<0.05, *t*-test). (**e**) Prepulse inhibition of the auditory startle response. Percentages by which 70-, 75- and 80-dB prepulse stimuli inhibited the startle response to the 120-dB stimulus (*n*=10 per genotype; *P<0.05, two-way repeated measures ANOVA). (**f**,**g**) Fear conditioning tests. (**f**) Classical fear conditioning. Freezing behaviour of conditioned mice was measured when exposed to the same environmental context 24 h after training and to a novel environment with the same tone (cue) 48 h after training (WT, *n*=10; KO, *n*=9; *P<0.05, *U*-test). Baseline freezing responses from the training session (before shock delivery) are shown for comparison. (**g**) Contextual memory retention. Freezing responses were measured in the training box or a differently textured new box 3, 7 and 28 days after the training (*n*=16 per genotype; *P<0.05, ***P<0.001, *U*-test). (**h**–**k**) Morris water maze test. (**h**) Schematic protocol of the Morris water maze test. Latencies to find the hidden platform are shown for the training session (**i**) and the probe tests (**j**) (WT, *n*=17; Lrfn2 KO, *n*=21; **P<0.01, *t*-test). (**k**) Time spent in the target or the other quadrants (mean of the three non-target quadrants) during the probe tests (WT, *n*=14; *Lrfn2* KO, *n*=17; *P<0.05, **P<0.01, ***P<0.001, paired *t*-test).

To determine whether the withdrawn *Lrfn2* KO mice also avoid inanimate or not, we performed marble burying assay, which are deemed to be rodent-specific repetitive and perseverative behaviours^31^,^32^. *Lrfn2* KO mice buried significantly more marbles than WT mice during the observation period (Fig. 3a). Because digging behaviour observed during the reciprocal social interaction test did not depend on genotype (Fig. 2c), the increased burying behaviour in KO mice indicates they had higher sensitivity to the marbles than did WT mice.

Similar tendency of *Lrfn2* KO was observed in wheel-running, which is considered a spontaneous and pleasurable activity with highly repetitive and stereotyped behaviour^33^. Compared to WT mice, *Lrfn2* KO mice exhibited excess repetitive running (Fig. 3b), and this hyperactivity was constantly observed over a week (Fig. 3c).

*Lrfn2* KO mice also exhibited an enhanced startle response to an acoustic stimulus (120 dB; Fig. 3d), and prepulse inhibition (PPI) of the auditory startle response was decreased in KO mice (Fig. 3e). The results indicated that *Lrfn2* KO mice have defective sensory information processing.

### Enhanced spatial learning and fear memory in *Lrfn2* KO mice

Intelligence quotient varies widely in ASD patients; it may be normal or above average^34^. We noticed enhanced memory in *Lrfn2* KO mice. In the classical fear conditioning test, the mice received two electrical footshock-tone pairings. *Lrfn2* KO mice showed robust freezing behaviour associated with contextual fear 1 h and 1 day after training, but a level comparable to that of WT mice in cue test that was carried out 2 days after training (Fig. 3f and Supplementary Fig. 3i). This led us to examine the duration of the enhanced contextual fear memory retention by measuring the freezing responses in the training box and in a differently textured novel box 3, 7 and 28 days after the training. *Lrfn2* KO mice froze numerically (but not significantly) more often than WT mice in the training box until day 7 but not on day 28 (Fig. 3g). When we compared the freezing responses in the two boxes, KO mice tended to discriminate the two boxes until day 7 (Fig. 3g and Supplementary Fig. 3i). By contrast, WT mice failed to discriminate them even at 3 days. There were no differences in basal or footshock-induced movement velocity (Supplementary Fig. 3i), indicating the intact nociception and motor function of *Lrfn2* KO mice. Taken together, these results indicate that fear memory is enhanced in *Lrfn2* KO mice.

Improved memory was also indicated by performance on the Morris water maze test. We carried out 2 days of massed learning consisting of four blocks of two training trials and a probe test that was carried out 1 day after the training trials (Fig. 3h,i). In the second probe test (Probe 2) after day 2 of training, *Lrfn2* KO mice reached the hidden platform faster than WT mice (Fig. 3j), although the time spent in the target quadrant did not differ between the genotypes (Fig. 3k). There were no differences in visual recognition, motor skills or motivation, as indicated by control experiments with visual platforms (Supplementary Fig. 3j–l). Thus, spatial learning performance is increased in *Lrfn2* KO mice.

### Altered excitatory postsynaptic proteins in Lrfn2 KO mice

Because both spatial learning and contextual fear memory retention depend on hippocampal function^35^,^36^, we next addressed the hippocampal phenotype in *Lrfn2* KO.

**Figure 4:**
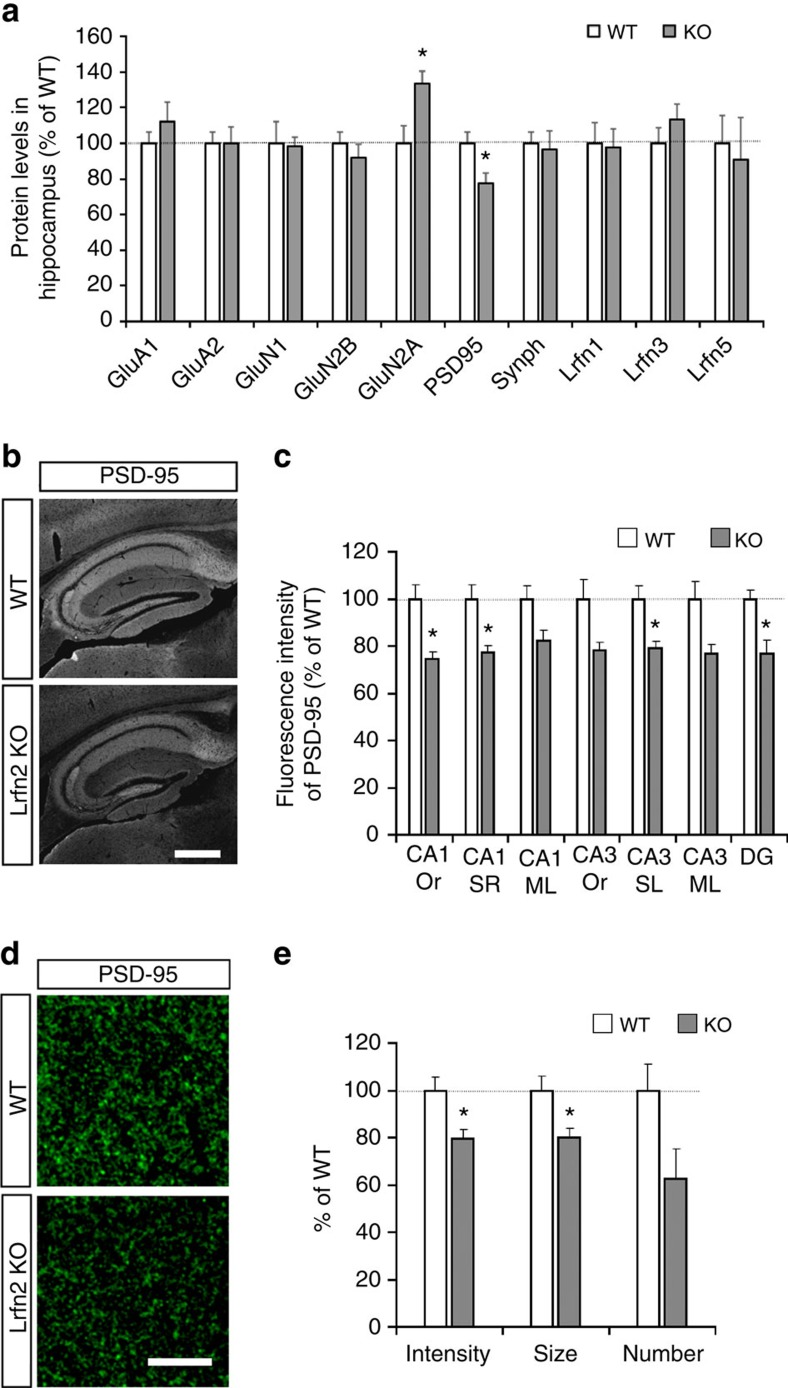
Quantification of synaptic proteins in *Lrfn2* KO mice. (**a**) Expression levels of synaptic proteins in the S1 fraction from adult hippocampus. The protein levels were densitometrically measured after immunoblotting (WT, *n*=8; KO, *n*=9 from three independent experiments; *P<0.05, *t*-test). (**b**) Immunostaining of hippocampus using an anti–PSD-95 antibody. Scale bar, 500 μm. (**c**) Quantification of fluorescence signal intensities in CA1, CA3 and dentate gyrus (DG) (*n*=3 mice, means of two images for each mice; *P<0.05, *t*-test). Or, oriens layer; SR, radial layer; ML, lacunosum molecular layer; SL, stratum lucidum layer. (**d**) Immunostaining for PSD-95 in the CA1 radial layer. Confocal images at a higher magnification than the images shown in **b**. Scale bar, 20 μm. (**e**) Quantification of intensity, size and number of PSD-95 puncta (*n*=6 sections from three mice per genotype; *P<0.05, *t*-test).

First, we used immunoblotting to examine the amounts of synaptic proteins in the hippocampus from 8-week-old mice, including GluA1, GluA2, GluN1, GluN2A, GluN2B, PSD-95, a presynaptic protein (synaptophysin) and other Lrfn proteins (Fig. 4a), and found that PSD-95 was significantly decreased and GluN2A was significantly increased. Immunofluorescence staining of the synapse markers showed lower PSD-95 in the CA1, CA3, and dentate gyrus regions of the KO (Fig. 4b,c) and no significant differences between the groups in synaptophysin, GluA1, GluA2 and GluN1 in these regions (Supplementary Fig. 4a–d). In the dorsal CA1 radial layer, both the intensity and size of the PSD-95–positive puncta were decreased by 20% (Fig. 4d,e), indicating that the absence of Lrfn2 resulted in reduced synaptic accumulation of PSD-95. In addition, the number of GluN2A puncta was comparable between WT and KO (Supplementary Fig. 4g), but the number of GluN2A puncta overlapping with PSD-95 signals was decreased (Supplementary Fig. 4h). Although the altered PSD-95 and GluN2A protein levels or localization, their mRNA levels were comparable between WT and KO (Supplementary Fig. 4i). These results raised the possibility that the loss of Lrfn2 causes abnormal structure and impaired function of excitatory synapses, because PSD-95 is an essential constituent of excitatory PSDs.

By contrast to the altered postsynaptic constituents, there were no significant differences in the intensity, size or number of Vglut1 (excitatory presynapse marker) or Vgat (inhibitory presynapse marker)-derived punctate immunopositive signals between WT and KO hippocampal CA1 radial layer (Supplementary Fig. 4e,f). The results indicated that the gross synapse numbers were comparable between WT and KO.

### Abnormal spine and synapse shapes in Lrfn2 KO mice

**Figure 5:**
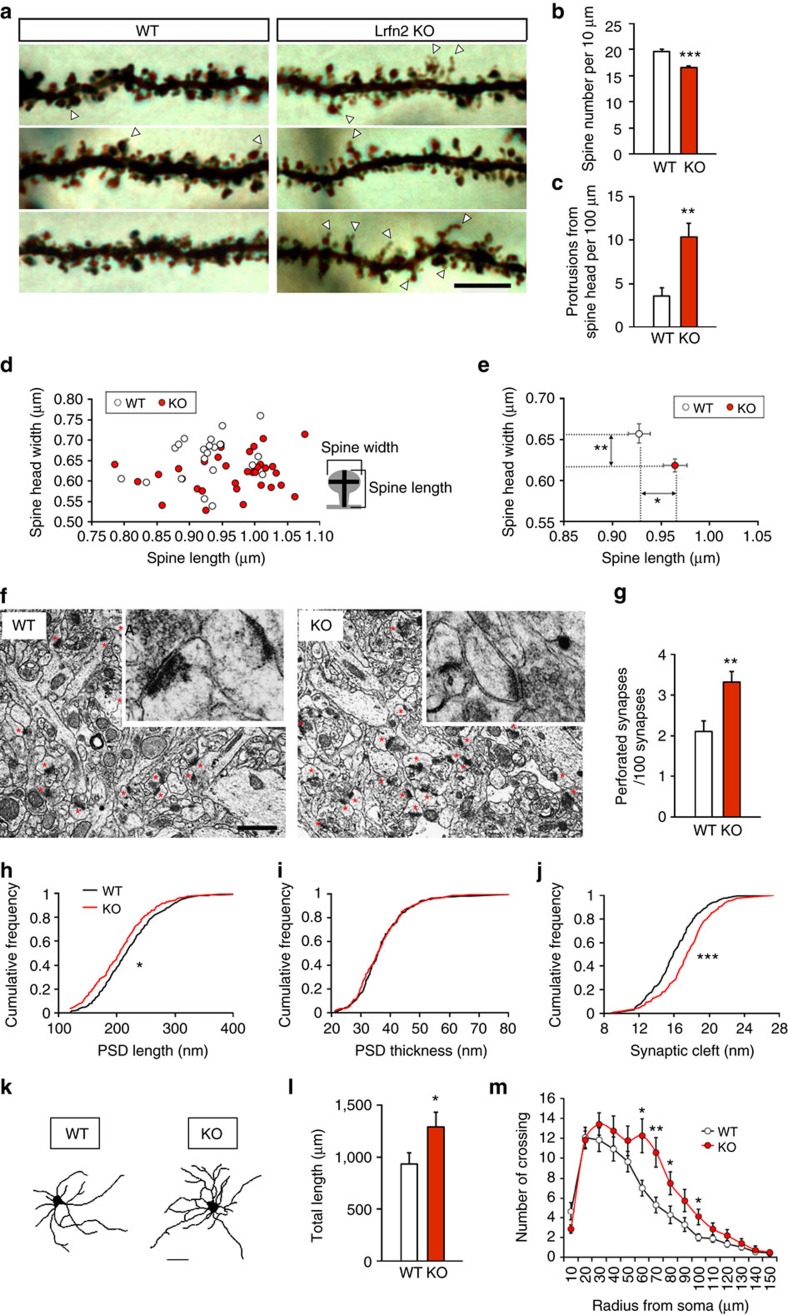
Morphological abnormalities of hippocampal synapses in *Lrfn2* KO mice. (**a**) Representative dendrite segments of Golgi-impregnated pyramidal neurons in the distal area of the hippocampal CA1 stratum radiatum from 8-week-old littermate WT and *Lrfn2* KO mice. Scale bar, 5 μm. (**b**) Spine densities of WT and *Lrfn2* KO neurons (WT, *n*=22 neurons from two animals, total 1,420 spines; KO, *n*=33 neurons from three animals, total 1,645 spines). *Lrfn2* KO mice showed a slight reduction in the spine density (***P<0.001, *t*-test). (**c**) Number of spines having a protrusion from the spine head. (**d**) Mean width of the spine head is plotted against mean spine length for each neuron. Data from 22 WT neurons and 33 neurons in *Lrfn2* KO. (**e**) Comparison of the mean of all spine widths plotted on the *y*-axis and the mean of all spine lengths on the *x*-axis (*P<0.05, **P<0.01, *t*-test). (**f**) Representative EM images of the hippocampal CA1 stratum radiatum. Red asterisks indicate synaptic contacts with a postsynaptic density, and insets show representative synapses at high magnification. Scale bar, 200 nm. (**g**–**k**) Quantitative analyses. The ratio of perforated synapses to total synapses (**g**) was larger in *Lrfn2* KO mice (WT, *n*=11 sections from three animals; *Lrfn2* KO, *n*=11 sections from three animals; *P<0.05, **P<0.01, *t*-test). (**h**–**j**) Cumulative frequency distribution of width of PSD length (**h**), PSD thickness (**i**) and synaptic cleft (**j**). Black lines indicate WT and red lines indicate *Lrfn2* KO (WT, *n*=306 synapses; KO, *n*=301 synapses; *P<0.05, ***P<0.001, K-S test). Similar results were obtained in two independent experiments. (**k**–**m**) Morphological analysis of the cultured hippocampal neurons. (**k**) Representative tracings of MAP-2–immunostained neurons from WT and *Lrfn2* KO mice at DIV10. (**l**) Quantification of the total dendrite length (WT, *n*=14 neurons; KO, *n*=12 neurons; *P<0.05, *U*-test). (**m**) The results of the Sholl analysis (WT, *n*=14 neurons; KO, *n*=12 neurons; *P<0.05, **P<0.01, *t*-test). Similar result was obtained in an independent experiment using DIV20 neurons (**k**–**m**).

Next we investigated the morphology of the spines on the apical dendrites of dorsal CA1 neurons. Golgi staining revealed that the shapes of spines in *Lrfn2* KO mice were clearly distinguishable from those in WT mice (Fig. 5a). When the total spine-like protrusions were counted, *Lrfn2* KO mice had a slightly but significantly lower number than WT mice (Fig. 5b). In contrast, we noticed a dramatic increase in the number of oddly shaped spines (Fig. 5c) with thin protrusions from the spine heads (arrowheads in Fig. 5a), reminiscent of ‘spinules'^37^. Morphometrical analysis of each neuron (Fig. 5d) confirmed that the mean length of spines was longer and the mean head width of spines was smaller in *Lrfn2* KO mice than in WT mice (Fig. 5e).

In parallel, we assessed the ultrastructure of the synapses by electron microscopy (EM) of the CA1 radial layer of the dorsal hippocampus (Fig. 5f). The ratio of perforated synapses to total asymmetrical synapses increased (*P*<0.01; *t*-test; Fig. 5g). We further quantified the morphology of the simple PSD synapses. The population of synapses in *Lrfn2* KO mice was shifted towards shorter PSD length (*P*<0.05; Kolomogorov–Smirnov [K–S] test; Fig. 5h), but PSD thickness was not changed compared to WT synapses (Fig. 5i). A cumulative plot of the width of the synaptic cleft showed that clefts in *Lrfn2* KO mice were shifted towards the wider direction (*P*<0.001; K–S test; Fig. 5j). Because the size of PSD is thought to correlate with the size of the spine head, the results of the EM analysis seemed to be consistent with the thin spines shown in the Golgi staining. Furthermore, the frequent appearance of oddly shaped, spinule-like spines and perforated PSD synapses implied an unstable spine structure, although no clear abnormalities were detected at the presynaptic terminal (Fig. 5f). Taken together, these results demonstrate that Lrfn2 plays an important role in maintaining normal spine and synapse structures *in vivo*.

We also investigated dendritic length and dendritic arborization in cultured hippocampal neurons stained with MAP2 after 10 days of culture (DIV10). Tracings of MAP2-immunoreactive dendrites (Fig. 5k) revealed that loss of Lrfn2 increased total dendritic length (Fig. 5l) and branching complexity as indicated by a Sholl analysis (Fig. 5m).

### Enhanced LTP and increased silent synapses in Lrfn2 KO mice

**Figure 6:**
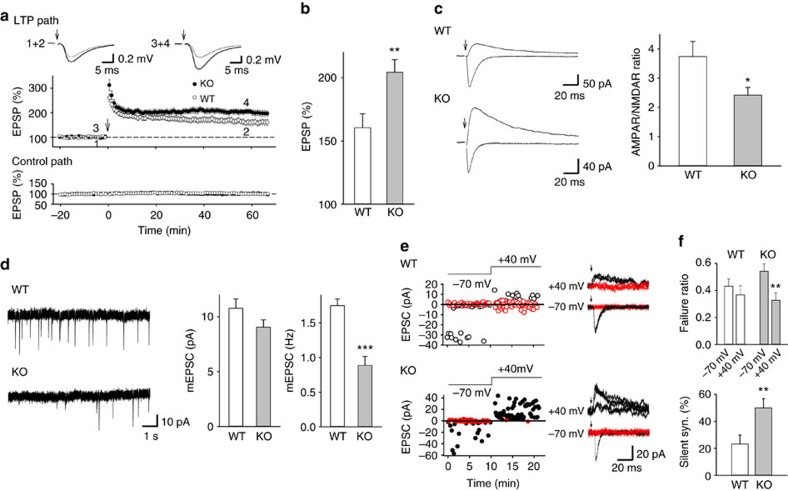
Regulation of LTP and the expression of synaptic AMPAR by Lrfn2. (**a**–**c**) Enhanced LTP and reduction of the AMPAR/NMDAR ratio in the hippocampus Shaffer collateral–CA1 synapses of *Lrfn2* KO mice. (**a**) LTP in the hippocampus. (upper) Representative averaged traces taken at the times indicated by numbers in the lower panel, and (lower) summary of time course of fEPSP slopes in LTP and control pathways. WT (open circles), *n*=15 slices from six mice; *Lrfn2* KO (filled circles), *n*=17 slices from seven mice. (**b**) Summary of LTP. LTP in *Lrfn2* KO mice was significantly enhanced (**P<0.01, *t*-test). (**c**) Summary of AMPAR/NMDAR ratios (WT, *n*=14 cells, 14 slices from four mice; *Lrfn2* KO, *n*=13 cells, 13 slices from five mice; *P<0.05, *t*-test). (**d**) Summary of mEPSC amplitude and frequency in WT (*n*=7 cell, seven slices from two mice) and KO mice (*n*=7 cell, seven slices from two mice) (***P<0.001). (**e**) Examples of AMPAR-EPSCs and NMDAR-EPSCs evoked by minimal stimulation in WT and KO mice. Red circles and traces indicate failed responses. (**f**) Summary of failure rates (upper) and per cent of silent synapses (lower) in WT (*n*=13 cells, 13 slices from three mice) and KO mice (*n*=21 cells, 21 slices from four mice) (**P<0.01). The recording was carried out using one mouse in a day. Mice in the same experimental group showed similar results.

Hippocampal synaptic plasticity is important for contextual memory and spatial learning^38^,^39^. Spine morphology is believed to reflect molecular composition, synaptic function and plasticity^40^,^41^,^42^. We next addressed whether the *Lrfn2* KO hippocampus shows electrophysiological changes in synaptic plasticity such as LTP. LTP of field excitatory postsynaptic potentials (fEPSP) at Schaffer collateral/CA1 synapses was significantly enhanced in *Lrfn2* KO mice compared to WT littermates (WT, 160.5%±10.9%; KO, 204.3%±9.9% of baseline at 60 min after tetanus; *P*<0.01; *t*-test; Fig. 6a,b). No significant differences were observed for either the paired-pulse (Supplementary Fig. 5a) or post-tetanic potentiation ratio (Supplementary Fig. 5b) between the two genotypes, suggesting that the probability of transmitter release in *Lrfn2* KO mice was normal. To further examine the postsynaptic alterations, we assessed the ratio of AMPAR- to NMDAR-mediated excitatory postsynaptic currents (EPSCs) of CA1 pyramidal neurons. *Lrfn2* KO mice showed significantly lower AMPAR/NMDAR ratios than WT mice (WT, 3.74±0.51; KO, 2.42±0.26; *P*<0.05; *t*-test; Fig. 6c).

When we evaluated spontaneous miniature EPSCs (mEPSCs), the mEPSC frequency was strongly reduced in *Lrfn2* KO hippocampus (WT, 1.75±0.10 Hz; KO, 0.89±0.13 Hz; *P*<0.001; *t*-test; Fig. 6d). Together with the decreased AMPAR/NMDAR ratios and intact excitatory synapse numbers, we hypothesized that a part of the excitatory synapses may have become non-functional at normal resting potential (silent synapse) in *Lrfn2* KO hippocampus. To test this idea, we carried out minimal stimulation experiments and assessed silent synapses, the ones that have only NMDARs^43^,^44^. Minimal stimulation evoked AMPAR-EPSCs at −70 mV and NMDAR- (plus AMPAR-) EPSCs at +40 mV with failures in both genotypes (Fig. 6e), however, the failure rate at +40 mV was significantly lower than that at −70 mV only in KO mice (WT, 0.43±0.05 at −70 mV, 0.37±0.07 at +40 mV; KO, 0.54±0.05 at −70 mV, 0.33±0.05 at +40 mV; *P*<0.01; Fig. 6f). We also calculated the percentage of the silent synapses by using a formula reported previously^43^ (see extended experimental procedures). As a result, the silent synapse was markedly increased in Lrfn2 KO hippocampus (WT, 23.2±6.7%; KO, 50.0±6.8%; *P*<0.01; *t*-test; Fig. 6f).

**Figure 7:**
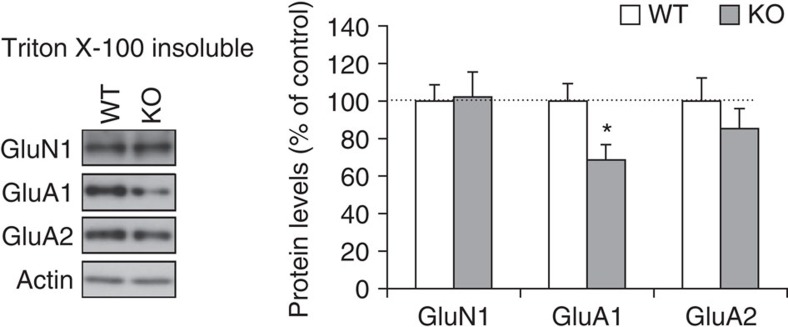
Immunoblot analysis of Triton X-100-insoluble fractions. Expression levels of synaptic proteins in 1% Triton X-100-insoluble fractions from adult hippocampus. The protein levels were densitometrically measured after immunoblotting (WT, *n*=8 mice; *Lrfn2* KO, *n*=9 mice; *P<0.05, *t*-test). Similar results were obtained in three independent experiments.

Trafficking of AMPAR at postsynaptic sites is involved in LTP^45^. We therefore quantified the amount of hippocampal GluA1, GluA2 and GluN1 proteins in the PSD-enriched fraction (Triton X-100 insoluble) by immunoblot analysis. The GluA1 content was significantly reduced in *Lrfn2* KO hippocampus without affecting the GluN1 content (Fig. 7 and Supplementary Table 1), whereas the content in the total cell lysate was not reduced. This finding indicates that an Lrfn2 deficit results in lower basal synaptic expression of GluA1-containing AMPAR. It seems that Lrfn2-deficient spines provide potential space for the addition of GluA1-containing AMPAR in the postsynaptic membrane during LTP.

### Lrfn2 enhances synaptic AMPAR incorporation with PSD-95

To gain more insight into the effects of Lrfn2 on AMPAR dynamics, we analysed surface expression of AMPAR and AMPAR-mediated currents in rat hippocampal neurons transfected with Myc-tagged Lrfn2; Myc-tagged Lrfn2ΔC, which cannot bind PSD-95 due to the deletion of two C-terminal amino acids in the PDZ-binding domain; and Flag-tagged PSD-95 (ref. 22).

**Figure 8:**
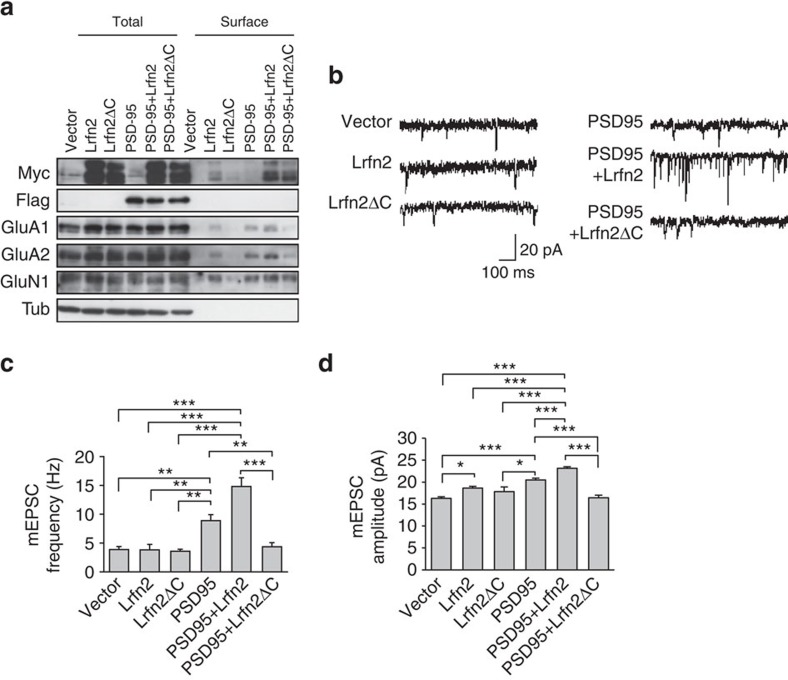
Interaction between Lrfn2 and PSD-95 facilitates the surface expression and synaptic strength of AMPAR. (**a**) Biotinylation assay of cultured rat hippocampal neurons overexpressing Myc-Lrfn2, Myc-Lrfn2ΔC, Flag-PSD95, the combination of Myc-Lrfn2 and Flag-PSD95, or the combination of Myc-Lrfn2ΔC and Flag-PSD95. Neurons were transfected with the indicated vectors at DIV10 and biotinylated at DIV17. Immunoblots showed both total and biotinylated (surface) proteins of glutamate receptors. (**b**) Examples of AMPAR-mediated mEPSCs recorded from cultured rat hippocampal neurons. Neurons were transfected with the indicated expression vectors at DIV10 and analysed at DIV20–24. (**c**,**d**) (**c**) Summary of mEPSC frequency (*n*=5 neurons for each transfection except Lrfn2ΔC and PSD95 [*n*=6 neurons each]; *P<0.05, **P<0.01, ***P<0.001, ANOVA and post hoc Tukey–Kramer test). (**d**) Summary of mEPSC amplitude (Vector, *n*=286 events from five neurons; Lrfn2, *n*=268 events from five neurons; Lrfn2ΔC, *n*=159 events from six neurons; PSD95, *n*=457 events from five neurons; PSD95+Lrfn2, *n*=678 events from five neurons; PSD95+Lrfn2ΔC, *n*=202 events from five neurons; *P<0.05, **P<0.01, ***P<0.001, ANOVA and post hoc Tukey–Kramer test). The results are obtained using four independently cultured neurons.

We first assessed plasma membrane expression of GluA1 and GluA2 in the transfected neurons with a surface biotinylation assay (Fig. 8a). Immunoblots for the biotinylated surface fractions showed that overexpression of Lrfn2 increased the surface expression of not only of endogenous GluN1 but also of GluA1 and GluA2, compared to that of the empty vector. In contrast, the transfected Lrfn2ΔC, which was excluded from the surface, did not elicit any changes in the receptors' surface expression. These results suggest that the PDZ-binding domain is required for the surface expression of both Lrfn2 and glutamate receptors. Overexpression of PSD-95 increases surface expression of AMPAR, leading to neuronal excitability in cultured hippocampal neurons^46^. Concordant with this, we observed increased surface expression of receptor subunits in our PSD-95–overexpressing neurons. Furthermore, co-expression of Lrfn2 with PSD-95 resulted in more membrane targeting of Lrfn2 and the glutamate receptors than did expression of Lrfn2 or PSD-95 alone. Co-expression of Lrfn2ΔC with PSD-95 increased surface expression of Lrfn2ΔC, but it selectively reduced the surface expression of GluA1 and GluA2 (but not GluN1) to the level of the control vector, indicating that the PDZ-binding domain mediates the membrane targeting and stability of AMPAR through PSD-95. In addition, Lrfn2ΔC seemed to preferentially interact with GluN1, excluding endogenous Lrfn2; this is consistent with the idea that Lrfn2 helps NMDAR trafficking due to the direct physical interaction between Lrfn2 and GluN1 via its extracellular or transmembrane domain^23^.

We also recorded miniature excitatory postsynaptic currents (mEPSCs) from the transfected hippocampal neurons (Fig. 8b–d). Neither Lrfn2- nor Lrfn2ΔC-overexpressing neurons showed a statistical difference in the averaged mEPSC frequency and amplitude compared with the control vector. PSD-95 transfection significantly enhanced the amplitude and frequency of mEPSCs, consistent with a previous report^46^. Furthermore, the transfection of Lrfn2 with PSD-95 resulted in an additional increase in both frequency and amplitude of mEPSCs compared to PSD-95 alone, indicating that the additional AMPAR targeted to synapses by exogenous Lrfn2 and PSD-95 are functional. In contrast, co-transfection of Lrfn2ΔC and PSD-95 resulted in mEPSCs comparable to those elicited by the control vector, even though PSD-95 was overproduced. This result indicates that interaction with the PDZ-binding domain is a critical process for the synaptic targeting of AMPAR, and Lrfn2ΔC seems to have a dominant-negative effect on endogenous Lrfn2. Taken together, these findings suggest that Lrfn2 functions at synapses together with PSD-95 and serves to target and stabilize AMPAR at postsynaptic sites.

### Functionally defective LRFN2 mutations in patients

**Figure 9:**
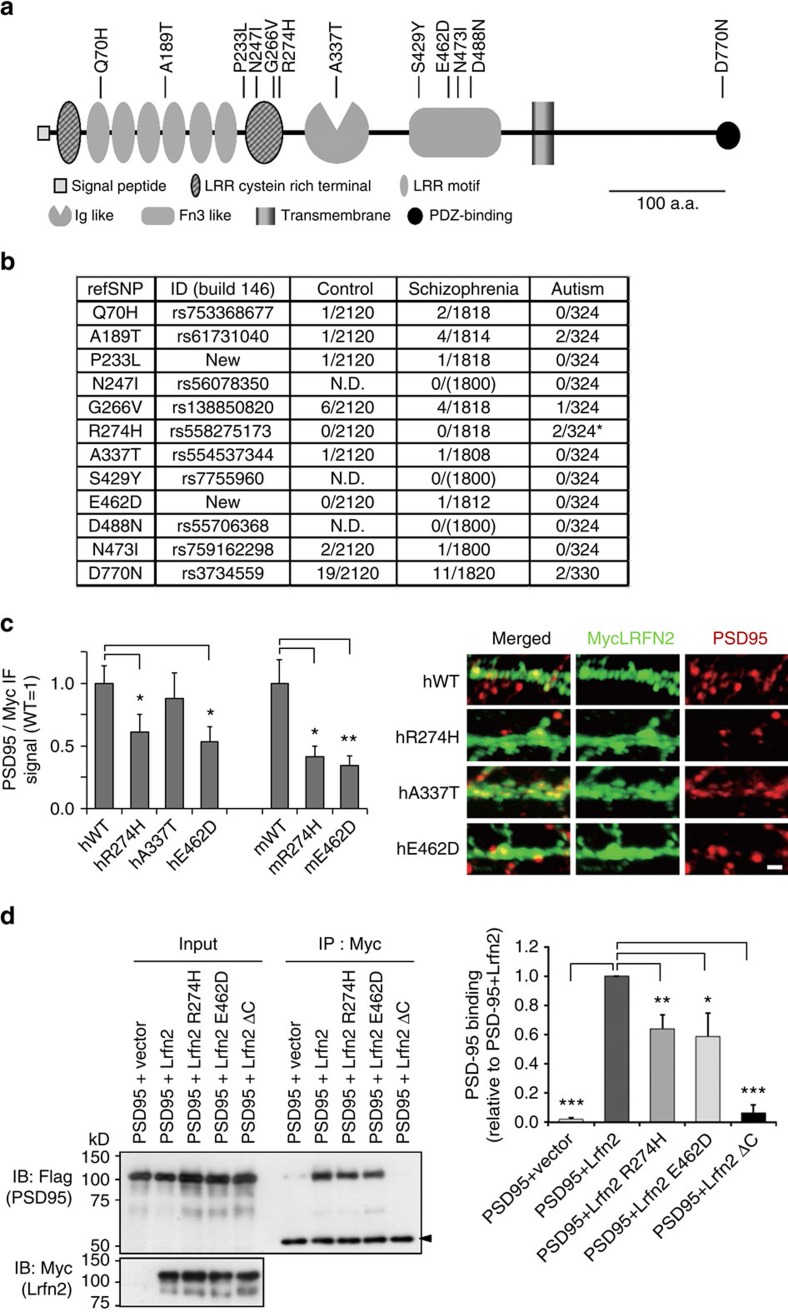
Resequencing analysis of the *LRFN2* protein-coding region from Asian patients with autism or schizophrenia. (**a**) Nonsynonymous SNPs on a schematic of the LRFN2 molecule. (**b**) Nonsynonymous SNPs in human *LRFN2* from patients with schizophrenia or autism and healthy controls. refSNP ID, the numbers indicated those deposited in the NCBI database and ‘new' indicates the newly identified nonsynonymous mutations in this study. The numbers of control, schizophrenia and autism columns indicate (allele number of patients with the mutations)/(allele number of total patients), where 0/(1,800) means the absence of the patients with corresponding mutations in the schizophrenia patients groups (*n*=1,820). Ambiguously genotyped subjects were excluded. Note that all carriers with mutations were heterozygotes. N.D., not determined. *P<0.05, Fisher's exact test. (**c**) Effects of the identified mutations of the subcellular localization of human LRFN2 (h) and mouse Lrfn2 (m) proteins. Co-localized PSD-95 signals (red) on those of Myc-tagged WT proteins or mutant proteins (green) with PSD-95 were quantified in cultured hippocampal neurons. Values for WT were adjusted to one (*n*=42–45 neurons from 15 images for each group; *P<0.05, **P<0.01, compared to WT, *t*-test). Similar results were obtained in three independent experiments. Right panels indicate the representative images (scale bar, 5 μm). Those for mouse proteins are indicated in Supplementary Fig. 8d. (**d**) PSD-95-binding capacities of WT and mutant mouse Lrfn2 proteins. Myc-tagged Lrfn2 proteins including Lrfn2ΔC were co-expressed with Flag-tagged PSD-95 in COS7 cells. Immunopreciptation was carried out using anti-Myc epitope antibody. Co-precipitated Flag-PSD-95 was detected with anti-Flag epitope antibody. Right graph indicates the densitometric quantification of the immunoblot (*n*=8 independent experiments; *P<0.05, **P<0.01, ***P<0.001, compared to WT, *t*-test).

Alterations in glutamatergic synapse structure and function represent a common underlying pathology in intellectual disability, ASD, schizophrenia^2^. The ASD-like behaviours of *Lrfn2* KO mice led us to conduct a pilot survey of mutations in human *LRFN2.* LRFN2 protein-coding exons and flanking introns were sequenced in DNA samples from 165 individuals with autism and 910 patients with schizophrenia and 1,060 mentally healthy controls. Patients with schizophrenia were included because of the partial genetic commonality in the etiologies of ASD and schizophrenia^46^,^47^ and the presence in *Lrfn2* KO mice of cognitive dysfunction typically found in mouse models of schizophrenia, such as the PPI deficit^47^. We detected several novel nonsynonymous single nucleotide polymorphisms (SNPs; Fig. 9). When we analysed the positions of the nonsynonymous SNPs identified in the current study and the known SNPs, we found two SNP clusters, one in the leucine-rich repeat C-terminal domain and the other in the FnIII domain (Fig. 9a). Eight out of the 12 nonsynonymous SNPs were located in positions highly conserved among vertebrates (Supplementary Fig. 6). The minor allele frequencies varied from 0.00047 to 0.0090, and the most common SNP was D770N, located near the C-terminus.

The allele frequencies of most of the nonsynonymous SNPs were not significantly different from those of healthy control subjects. However, R274H was found only in the patients with autism (allelic *P*=0.018 by Fisher's exact test; Fig. 9b). Although R274H was identified in nonsymptomatic consanguineous family members (Supplementary Fig. 7a), it may be worth considering its involvement in ASD pathogenesis in light of the strong evolutionary conservation of R274 (Supplementary Fig. 6). In addition, several other SNPs across the gene displayed nominally significant transmission distortion in a Chinese cohort (Supplementary Table 2). As a rare variant, we found E462D only in individuals with schizophrenia (Fig. 9b), which also occurs in an evolutionarily conserved position (Supplementary Fig. 6). The amount of LRFN2 transcript in the postmortem brains of individuals with schizophrenia was comparable to that of control subjects (Supplementary Fig. 7b).

When we quantified PSD-95 co-localized on LRFN2 R274H, A337T, E462D or WT-transfected hippocampal neurons by immunofluorescence staining, the signals on the R274H and E462D transfectants were significantly lower than that of WT, and the differences were recapitulated in mouse Lrfn2 R274H and E462D equivalent mutants (Fig. 9c and Supplementary Fig. 8d,e). In immunoprecipitation assay, both Lrfn2 R274H and Lrfn2 E462D co-precipitated less PSD-95 than Lrfn2 WT (Fig. 9d). We also noticed that the dense signals on cell body were more frequent in E462D transfectants (Supplementary Fig. 8a,b), which may be caused by trafficking abnormality (Supplementary Fig. 8c). These results indicated the presence of R274H and E462D impaired the molecular function of LRFN2.

## Discussion

LTP was enhanced in CA1 pyramidal neurons in *Lrfn2* KO hippocampal slices. LTP induction involves the passage of calcium through NMDAR following activation of Ca^2+^/calmodulin-dependent protein kinases II and the resultant recruitment and insertion of AMPAR into the postsynaptic membrane^48^. Overexpression of Lrfn2 and PSD-95, an Lrfn2-binding protein, resulted in augmentation of AMPAR-EPSCs and increased surface expression of AMPAR compared to the excitability generated by PSD-95 alone (Fig. 8). Because overexpression of Lrfn2 alone did not cause any apparent changes, regulation of synaptic efficacy by Lrfn2 may depend on the expression level of PSD-95. On the other hand, the combination of PSD-95-binding–deficient Lrfn2ΔC and PSD-95 selectively inhibited AMPAR current/surface expression. Therefore, the Lrfn2–PSD-95 association is essential for PSD-95 to exert its role as a regulator of AMPAR dynamics. The role of Lrfn2 in this regard seems to fit with the concept of a ‘slot protein'^49^,^50^, which serves as the receptor binding site and determines the number of surface AMPAR.

The analysis of *Lrfn2* KO mice supported the presumptive role of Lrfn2 in the recruitment and subsequent stabilization of AMPAR at synaptic sites. First, we found a reduction of the AMPAR/NMDAR ratio (Fig. 6c), increment of silent synapse (Fig. 6e,f) and less GluA1 protein in the Triton X-100-insoluble fraction (PSD-enriched fraction) in *Lrfn2* KO hippocampus (Fig. 7). Because synapses in *Lrfn2* KO mice possess less AMPAR, there is more room for AMPAR to be inserted at postsynaptic sites, and synaptic transmission can be potentiated more^48^. Second, immunoblot and immunostaining analyses showed a significant reduction of PSD-95 protein levels and the clustering of PSD-95 protein in the radial layer of CA1 pyramidal cells. These two results suggest that the absence of Lrfn2 results in decreased synaptic PSD-95, followed by destabilized retention of AMPAR at the synapse.

Collectively, Lrfn2 may regulate the surface AMPAR fraction through its interaction with PSD-95. We propose that this property of Lrfn2 largely accounts for the enhanced LTP in the KO mice. In agreement with this idea, PSD-95–deficient mice consistently show enhanced LTP^14^,^15^,^51^ and exhibit more synapses that lack AMPAR (increased silent synapses)^15^.

While the interaction between Lrfn2 and PSD-95 can explain the reduced surface AMPAR and the enhanced synaptic plasticity in *Lrfn2* KO mice, increment of GluN2A amount and reduced PSD-95 overlapping GluN2A puncta in *Lrfn2* KO hippocampus remains to be clarified. However, at this point, we speculate that following two mechanisms could be involved in the GluN2A abnormalities. First, the reduced PSD-95 in *Lrfn2* KO may have altered the GluN2A protein dynamics because GluN2A interacts directly with PSD-95 and PSD-95 increases GluN2A surface expression by regulating insertion and internalization of NMDAR (reviewed in ref. 52). Second, the absence of GluN1-binding activity of Lrfn2 (ref. 25) may have influenced the GluN2A protein dynamics because GluN1 plays crucial role in the release of GluN2A from endoplasmic reticulum^53^. In either explanation, regulatory mechanism underlying GluN2A re-distribution and metabolism may be needed for better understanding.

Besides the enhanced synapse plasticity in *Lrfn2* KO mice, our results shed new light on the roles of Lrfn2 in controlling neuronal morphology. First, the altered spine shape in the *Lrfn2* KO mice was similar to that in PSD-95 KO mice^15^ in that spine length was significantly increased. Furthermore, the role of PSD-95 in spine enlargement and stabilization has also been shown in overexpression^46^ and RNAi knockdown^12^ experiments. Therefore, the morphological changes in *Lrfn2* KO synapses may partly reflect the reduced PSD-95 protein level. There is a close correlation between spine head size and PSD size^54^ and between PSD size and AMPAR/NMDAR ratio^55^. These facts are very consistent with the reduction of spine width, PSD length and AMPAR/NMDAR ratio in *Lrfn2* KO.

Second, we saw oddly shaped thin protrusions from spines that seemed different from the filopodia that emerge from the surface of the dendritic shaft. Rather, the protrusions from the oddly shaped spines in *Lrfn2* KO mice are similar to so-called synaptic spinules or actin-based protrusions from spine head/SHP (spine head protrusion). On EM, these can be seen to project from the perforation of the PSD or at the edge of the PSD on the spine head and to invaginate into the presynaptic terminal^39^. In support of this similarity, the number of perforated synapses with split PSD increased in *Lrfn2* KO hippocampus (Fig. 5g). Postsynaptic perforations and spinules on spines increase after the high-frequency stimulation that induces LTP^56^,^57^, and their presence implies dynamic synaptic remodelling or coordination that depends on synaptic activity. Lrfn2 may maintain the intact spine shape by decreasing the spontaneous motility of spines.

Third, the widening of the synaptic cleft implies that the absence of Lrfn2 results in the weakening of synaptic adhesion *in vivo*. This defect may reflect the loss of the adhesive function that Lrfn2 provides through its interaction with presumptive presynaptic molecules, as seen in the absence of other synaptic adhesion molecules^6^,^18^,^19^, or it may occur indirectly through the *cis*-interaction of Lrfn2 with other Lrfn proteins^58^.

Last, the cultured *Lrfn2* KO hippocampal neurons showed increased dendrite length and dendritic arborization. We emphasize again the functional relevance to PSD-95, because PSD-95 knockdown also results in increased primary and secondary dendrite number^59^. Taken together, we speculate that Lrfn2 regulates neuronal structure at both spines and dendrites *in vivo*.

Above abnormalities in synaptic function and morphology may underlie the behavioural abnormalities in *Lrfn2* KO mice. In our behavioural test battery, *Lrfn2* KO mice exhibited social withdrawal, a tendency to perseverate and impaired communication skills, which are all included among the core symptoms of autism, together with sensory dysfunction and a tendency to depression, which are common comorbid symptoms of autism^60^. These phenotypes are fairly common among known autism model mice^30^. We therefore consider that the behavioural abnormalities in *Lrfn2* KO mice may partly mimic the pathophysiological status of autism patients. Furthermore it is tempting to speculate the relationship between the social withdrawal in *Lrfn2* KO and the antisocial personality disorder which is associated with a locus near *LRFN2* (ref. 26). While we clarified the defective synapse morphology and function in *Lrfn2* KO hippocampus, the neural circuit basis of the defective social behaviour in *Lrfn2* KO is still an open question.

On the other hand, the enhanced spatial learning and fear memory seem to be behavioural phenotypes unique to *Lrfn2* KO mice. Interestingly, an LRFN2-happloinsufficiency patient showed an enhanced long-term memory with speech delay^25^, suggesting the common role of the gene in humans. Memory function is also enhanced in a narrowly restricted domain in a subset of ASD patients (‘savant syndrome')^61^. The enhanced memory function of *Lrfn2* KO could reflect the altered cognitive function of ASD patients, although it is premature to conclude this definitively.

Because Lrfn2 protein levels in the hippocampus and cerebral cortex increase during postnatal development, Lrfn2 may be a key component of excitatory synapse maturation in terms of increasing the AMPAR/NMDAR ratio, spine head size and PSD size. The role of Lrfn2/SALM1 in synapse maturation is well contrasted with excitatory synapse number regulation by Lrfn4/SALM3 and Lrfn3/SALM4. Lrfn4/SALM3 KO mice show normal hippocampal CA1 synaptic morphology and plasticity, and normal memory functions whereas the excitatory synapse density is decreased^62^. SALM4 negatively regulates excitatory synapses via *cis* inhibition of SALM3 (ref. 63). Thus the Lrfn family proteins cooperatively regulate synapse formation and synapse maturation. Together with the profound cognitive function deficits, we believe that *Lrfn2* KO is a model for neurodevelopmental disorders that conforms to the synapse maturation defect as a cause of ASDs. Furthermore, the behavioural abnormalities of the *Lrfn2* KO and the functionally impaired *LRFN2* missense mutation warrant more extensive genetic analyses of *LRFN2* using larger samples and different populations.

## Methods

### Animals

All animal experiments were approved by the institutional ethical committees and carried out in accordance with the guidelines for animal experimentation in the Nagasaki University Graduate School of Biomedical Sciences, Gunma University Graduate School of Medicine, and RIKEN.

### Generation of Lrfn2 KO mice

A BAC clone containing *Lrfn2* was purchased from BACPAC resources of the Children's Hospital Oakland Research Institute. An *Lrfn2* targeting vector was constructed to replace exon 2, which contains the ATG translation initiation codon, with a neomycin resistance gene cassette flanked by a loxP sequence (Neo). Homologous genomic DNA with the Neo cassette was joined with a diphtheria toxin—a cassette for negative selection. Linearized targeting vectors were electroporated into E14 ES cells, and homologous recombinants were isolated by G418 selection. The ES clones were screened by Southern blot analysis. Correctly targeted ES clones were injected into blastocysts of C57BL/6J mice, which were then used to produce chimeric mice. After confirmation of germline transmission, the Neo cassette was removed by crossing mice that had germline transmission with transgenic mice expressing Cre recombinase in germ cells^64^. The Cre recombination was confirmed by PCR and Southern blot analyses. *Lrfn2*-heterozygous (*Lrfn2*^*+/−*^) mice were backcrossed into the C57BL/6J inbred background for at least six generations before the behavioural experiments were started. Mutant animals were genotyped by PCR using DNA and the following primers: forward primer F1 for WT and KO allele (5′-CACATGGTGCGTGCAATTTAGG-3′); Reverse primers R1 for WT allele (5′-GGCAATTGCCCTTATCAAGAGC-3′) and R2 for KO allele (5′-TTGAGTAAGAGCAAGAACCCAGC-3′). Alternatively, the mutant animals were genotypes by Southern blot analysis after *Bgl*II and *Eco*RI digestion of genomic DNA with 5′ and 3′ probes.

### Antibodies

The following antibodies were used: GluA1 (for immunoblot, 1:400, PC246, Calbiochem; for immunostaining, 1:200, SC-13152, Santa Cruz), GluA2 (1:500 for immunoblot, 1:200 for immunostaining, MAB397, Chemicon), GluN1 (1:2,000 for immunoblot, 1:200 for immunostaining, 114011, Synaptic Systems), GluN2A (for immunoblot, 1:1,000, 1500-NR2A, PhosphoSolutions; for immunostaining, 1:300, AB_2571605, Frontier Institute), GluN2B (1:200, 610416, BD Biosciences), PSD-95 (for immunoblot, 1:1,000, 05-494, Upstate or 1:2,000, 75-028, NeuroMab; for immunostaining, 1:400, 75-028, NeuroMab), Synaptophysin (1:4,000 for immunoblot, 1:200 for immunostaining, S-5768, Sigma), Synapsin I (1:500, AB1543P, Chemicon), Vglut1 (1:300, 135302, Synaptic Systems), Vgat (1:500, 131003, Synaptic Systems), TGN38 (1:500, 2F7.1, 804095, Alexis), tubulin (1:1,000, G7121, Promega), actin (1:1,500, A2066, Sigma), GFP (1:1,000, 598, MBL), Myc (1:1,000, 9E10, SC-40, Santa Cruz), Flag (1:2,000, M2, F3168, Sigma), HA (1:500, 12CA5, 11583816001, Roche; 1:500, 3F10, 11867423001, Roche). Rabbit polyclonal antibodies to Lrfn family proteins were raised against peptides as follows: Lrfn2N, APPLSFSFGGNPLHC; Lrfn2C (-ESTV), CARGTFGSSEWVM; Lrfn2C, CEESDLVGARGTFGSSEWVMESTV; Lrfn1, CGDLGLGSARARLAFTSTEWML; Lrfn3, CEPWGPSHEPAGP; Lrfn4, CRGVGGSAERLE; Lrfn5, CTNVDQNVQETQRLESI. Antisera were affinity purified on SulfoLink columns (Pierce) that were coupled with antigen peptides following the instructions of the manufacturer.

### DNA constructs

Mouse Lrfn proteins and PSD-95 expression vectors were previously described^24^. The wild-type LRFN2 expression vector was generated by inserting human LRFN2 protein-coding regions PCR amplified from the human brain cDNA (Clontech) and cloned into the pGEMT easy plasmid vector (Promega). PCR-based site-directed mutagenesis was carried out using appropriate primers for R274H, A337T and E462D. The cloned fragments were sequenced and subsequently PCR modified to have a *Bgl*II site just downstream of the predicted signal peptide cleavage and a *Not*I site just downstream of the termination codon. The generated fragments were inserted into N-terminally epitope-tagged pSecTag2 vectors^24^.

### Biochemical analysis

For the protein preparation from mouse brains (Fig. 1), whole or parts of brains were homogenized in RIPA buffer (50 mM Tris-HCl [pH 7.5], 150 mM NaCl, 1% NP-40, 0.5% SOD, 0.1% SDS, 10 mM EDTA) supplemented with complete protease inhibitor cocktail (Roche), and cell debris was removed by centrifugation at 12,000*g* at 4 °C for 30 min. Proteins for the antibody checking, subcellular fractionation analysis and immunoprecipitation analysis (Supplementary Fig. 1) were prepared as described^65^. For quantification of hippocampal proteins (Fig. 4a), hippocampi from 8-week-old WT mice (*n*=8) and *Lrfn2* KO mice (*n*=9) were individually collected, followed by homogenization and centrifugation (1,000*g*, 10 min) to obtain the S1 fraction. SDS was added to a portion of the S1 fraction to a final concentration of 1%, and the solution was then agitated for 1 h at 4 °C and sonicated. For preparation of the Triton X-100-insoluble fraction (Fig. 7), P2 crude synaptosomal fractions were pelleted by centrifugation (10,000*g*, 30 min) of the remaining S1, which was then dissolved in an equal volume of TET buffer (1% Triton X-100, 2 mM EDTA, 20 mM Tris-HCl, pH 7.5) supplemented with the protease inhibitor cocktail and agitated for 1 h at 4 °C. After centrifugation at 100,000*g* for 1 h, the pellet was resuspended in 0.1 vol (of the original S1) of 1% SDS, 2 mM EDTA, and 20 mM Tris-HCl, pH 7.5; 0.9 volume of TET buffer was added, and the solution was agitated for 1 h at 4 °C.

For immunoblot analysis, equal amounts of protein extracts were separated by SDS-PAGE and transferred onto PVDF Immobilon-P membranes (Millipore). The membranes were blocked with 5% skim milk in TBST (20 mM Tris-HCl [pH 7.5], 150 mM NaCl, 0.1% Tween-20) and probed with specific antibodies, followed by peroxidase-conjugated secondary antibodies. Signals were developed using the ECL Plus reagents (GE Healthcare) and exposed to Hyperfilm ECL (GE Healthcare). Densitometric analysis of bands was performed using ImageJ software (http://rsbweb.nih.gov/ij/). Total net signal (total signal—background) for each animal was normalized against the corresponding signal for actin or tubulin, and the average signal was calculated for each genotype.

For immunoprecipitation, the synaptosomal fraction (250 μg) from a mouse brain without the cerebellum was incubated in RIPA buffer for 1 h at 4 °C. The lysates were obtained by centrifugation for 20 min at 12,000*g* and precleaned with protein A-agarose, then incubated with anti-Lrfn2 antibody or normal rabbit IgG of the same quantity overnight at 4 °C. After incubation with protein A-agarose, the agarose beads were precipitated and washed with RIPA buffer five times, and then subjected to the immunoblot analysis.

For mRNA level analysis, RNA was isolated from Lrfn2 WT and KO hippocampus with Trizol Reagent (Thermo Fisher). cDNA was synthesized with SuperScript II Reverse Transcriptase (Thermo Fisher). Realtime RT-PCR analysis was carried out using Power SYBR Green PCR Master Mix (Thermo Fisher), ABI PRISM 7900HT (Thermo Fisher), and following primers (GluN2A, 5′-ATTCAACCAGAGGGGCGTA-3′ and 5′-TTCAAGACAGCTGCGTCATAG-3′; GluN2B, 5′-CCCTAGGCATGTGACTTGAAA-3′ and 5′-CACACACACACGCACACG-3′; PSD-95, 5′-CACCCTATCGCCATCTTCA-3′ and 5′-GCTCCTCTGTGATCCGCTTA-3′; Lrfn2, 5′-GGGTCGCTACTTTTGGCATA-3′ and 5′-GCAGCTTGTGTGTGTGCTG-3′).

### Behavioural testing

Adult *Lrfn2* KO and WT mice (2- to 6-month-old male littermates from mated heterozygotes) were used for behavioural tests unless otherwise noted. All mice were male, 2–3 months of age at the start of behavioural testing, and were tested beginning with less stressful behavioural tasks. Mice were housed on a 12:12 h light:dark cycle, with the dark cycle occurring from 20:00 to 8:00; behavioural experiments were carried out between 10:00 and 17:00.

### Classical fear conditioning

A fear conditioning test was conducted for 3 consecutive days. This test consisted of three parts, a conditioning trial (Day 1), a context test trial (Day 2), and a cued test trial (Day 3). For fear conditioning on Day 1, mice were placed in a novel rectangular chamber with a grid floor (34 × 26 × 30 cm). After a 2-min baseline period, 2 tone-shock pairings were presented. Each pairing consisted of a 20-s, 85-dB, 2,800-Hz tone ending simultaneously with a 2-s, 0.75-mA shock. There was a 1-min interval between each pairing. The mice remained in the chamber for 1 min after the second shock before being returned to the home cage. The total duration of training was 500 s. A context test was performed in the same conditioning chamber for 3 min in the absence of the white noise 24 h after the conditioning trial. Further, a cued test was performed in an alternative context with distinct cues; the test chamber was different from the conditioning chamber in terms of colour, floor structure (no grid), and shape (triangular). The cued test was conducted 24 h after the contextual test was finished and consisted of a 2-min exploration period to evaluate nonspecific contextual fear followed by a 2-min conditioned stimulus (tone without foot shock) to evaluate the acquired cued fear. The percentage of the freezing response (immobility excluding respiration and heartbeat) duration in each time bin was measured as an index of fear memory. Data were collected and analysed using Image J FZ2 (O'Hara, Tokyo, Japan).

### Morris water maze

The water maze apparatus was composed of a circular pool (1.0-m diameter) filled with opaque water (25 °C) and an escape platform (10-cm diameter), which was submerged 1 cm below the water surface. The training protocol consisted of 2 days of four blocks (two training trials per block). The swim patterns were monitored by computer-linked overhead video camera, and the escape latency to the platform was recorded. We carried out two probe tests. The first test was done at the end of the third session and the second at the end of the last session. During the probe test, the platform was removed and the mice were allowed to swim in the pool for 60 s. The time spent in each quadrant was recorded. The movements of mice in the maze was recorded and analysed with Image J WM (O'Hara).

### Open field locomotion

Spontaneous activity in the open field was tested in an open field apparatus (50 × 50 × 40 cm; O'Hara). Each mouse was placed in the centre of the open field and was allowed explore for 15 min. The behaviour was recorded by a computer-linked overhead video camera. Data were collected and analysed using Image J OF4 (O'Hara).

### Home cage activity measurement

Spontaneous activities of mice in the home cage were measured by a 24-channel ABsystem 4.0 (Neuroscience, Tokyo, Japan) for 6 days. Cages were individually set into compartments made of stainless steel in a negative pressure rack (JCL, Tokyo, Japan). A piezoelectric sensor on the ceiling of each compartment detected movements of the mice after habituation to the cages for 1 day.

### Light/dark transition test

The apparatus used for the light/dark transition test consisted of a cage (20 × 40 × 20 cm) divided into two sections of equal size by a partition containing a door (O'Hara). One chamber was brightly illuminated (250 Lux), while the other chamber was dark (0 Lux). Mice were placed in the light box and allowed to move freely between the two chambers with the door open for 10 min. The total number of transitions, per cent time spent in the light side, and per cent distance travelled in the light side were recorded and calculated by ImageJ LD4 (O'Hara).

### Wheel-running activity

Mice were housed individually in cages (24 × 11 × 14 cm) equipped with a steel running wheel (O'Hara). Wheel-running activity (3 counts per rotation) was detected by a wheel-metre and recorded every 10 min by an online PC computer system (WW-3302, O'Hara). The mouse was able to move freely in the wheel-cage and was given free access to solid food and water during the test. The data from the initial 10 days were omitted from those of total 17 days recording.

### Reciprocal social interaction test

Ten male WT and 10 *Lrfn2* KO mice (residents) were housed individually for 4 weeks, and 20 male DBA2 mice (intruders) were housed in groups of five mice. The home cages of the residents were not changed for at least 3 days before testing. Reciprocal social interaction tests were performed on 3 consecutive days. On each day, the test was started by placing an unfamiliar intruder in the home cage of a singly housed resident, and their behaviours were recorded on digital video for 5 min. Social responses observed in the resident were scored by the time spent in active social investigation (approaching, sniffing), digging, non-social moving, resting and social avoidance (escaping and hiding under bedding material). The scores for each behaviour were averaged for each genotype.

### Three-chamber social approach test

Three-chamber sociability test apparatus was a rectangular, three-chambered box [both side chambers 20 × 15 × 25 (height) cm; centre chamber 20 × 10 × 25 (height) cm], made from a white acrylic box with removable floor. The dividing walls have a 10 cm opening in the centre and the test mice were allowed to access from the centre chamber into the both sides of chambers. The apparatus was washed and cleaned with 70% ethanol between trials. Before the test session, the test mice were placed in the centre chamber and allowed to habituate all chambers for 10 min. For test session, an unfamiliar DBA2 male (stranger) was placed in a small, transparent cylinder (8 cm diameter × 15.5 cm height) with multiple holes (1 cm diameter each) spaced over the entire surface 1 cm∼ apart, that allowed olfactory, visual, auditory and tactile contacts but prevented deep contact such as direct interaction. The cylinder with unfamiliar mouse was placed in one of the side chambers and the empty identical cylinder was placed in the opposite chamber. The location of stranger was altered between trials. The test was started by placing one of the test mice in the centre chamber. The test mice were allowed to explore the three chambers freely for 5 min. The time spent in each chamber was measured using open field monitoring system (O'Hara) linked overhead video camera and the number of nose contact to each cylinder was counted manually.

### Ultrasonic vocalization analysis

USV were recorded in a soundproof box with a condenser ultrasound microphone (CM16/CMPA, Avisoft Bioacoustics, Berlin, Germany) connected to an amplifier/digitizer (Avisoft UltraSoundGate416H, Avisoft Bioacoustics). The recorded files were transferred to sound analysis software SASLab Pro Recorder Software (Avisoft Bioacoustics) for fast Fourier transform (512 FFT-length, 100% frame size, Hamming window, 50% time-window overlap). The number of calls, mean duration of each call, and peak frequency of each call were analysed. For the measurement of infant isolation calls, both male and female pups born from eight dams were tested at postnatal day 7. The home cage with a dam and her pups were moved to the experimental room at least 30 min before the recording. In the test session, the dam was removed and the pups were maintained at 35 °C in the home cage. Each pup was placed on a cotton pad in a plastic beaker (10 cm diameter, 11 cm height) and set in the soundproof box. USV were recorded for 5 min at a sampling frequency of 300 kHz. For measurement of ultrasonic courtship vocalizations, we used 15-month-old male mice that had been group-reared without exposure to females after weaning and three 1-month-old female mice that were assessed for oestrous phase on the day of experimentation. The male mice were exposed to an oestrous female mouse for 10 min once a day for 5 successive days before the recording. On the test day, the vocalizations were recorded during a 5-min exposure to an oestrous female in a home cage where the microphone was positioned above the cage. We confirmed that the male mice gave no vocalizations in the absence of a female before the tests.

### Marble burying test

Transparent plastic cages (25 × 18 × 15 cm) were filled with white paper bedding material (Paperclean, Japan SLC) to a 4-cm depth. Mice were placed individually in the test cages for 20 min (habituation trial) and then returned to their home cages. Fifteen blue glass marbles were evenly set at 5-cm intervals on the surface of the bedding material in the habituated cages. Then the mice were again placed in the habituated test cage for 20 min (test trial). During both trials, the test cage with the mouse was set in the infrared beam apparatus to measure activity. After 20 min, marbles that were more than two-thirds covered with paperclean were counted as buried marbles.

### Acoustic startle response and prepulse inhibition

Mice were habituated in their home cages for 1 h to 65-dB white noise. They were then placed into standard startle chambers. Each session was initiated with a 5-min acclimation period of white noise at 65 dB followed by 10 successive 120-dB tones to elicit the startle response (40 ms). Nine different trial types were then presented: 70, 75, 80, 85, 90, 95, 100, 110 or 120 dB (40 ms) with background noise at 65 dB. Each trial was presented five times, and the average response to each trial calculated. Immediately after the startle response trials, the PPI session was begun. During each PPI session, a mouse was exposed to the following types of trials: the omission of stimuli (no-stimulus trial); startle-alone trial (120 dB); three prepulse combinations (prepulse-pulse trials) using three prepulse intensities: 70, 75 and 80 dB. Each PPI session consisted of 10 presentations of each type of trial. PPI was assessed for each animal as a percentage (%PPI): 100 × (mean startle to pulse alone—mean startle to prepulse-pulse)/mean startle to pulse alone. The apparatuses and software used for data analysis were commercially available (Mouse Startle; O'Hara).

### Rotarod test

The rotarod (O'Hara) is a rotating rod that was accelerated from 4 to 40 r.p.m. over the course of 5 min. Individual mice was placed on the rod, and once they were balanced, the rod accelerated. The time at which the mouse fell from the rod was recorded. Each mouse went through four consecutive trials.

### Elevated plus-maze test

Each mouse was placed on the central square, facing an open arm of the plus-maze (with a central 5 × 5 cm platform, two open arms of 25 × 5 cm, two enclosed arms of 25 × 5 × 15 cm, O'Hara). Behaviour was recorded by an overhead video camera during the 5-min test period. Data analysis was performed automatically by ImageJ EPM software.

### Hidden-cookie test

Mice were first habituated to butter cookies in the home cage overnight. The next day, the mice were deprived of food for 24 h. The test was conducted in a new cage. A piece of butter cookie (0.7 mg per piece) was placed in a randomly chosen area on the cage floor, and then the entire cage floor was covered with corncob bedding to a depth of 4 cm. The subject was then placed into the cage, and latency to find the cookie was recorded up to a maximum time limit of 15 min. We defined finding the cookie as when the mouse held it in both paws.

### Histology and morphological analysis

For immunofluorescence staining of cryostat sections, the brains were cryoprotected with 30% sucrose after perfusion-fixation with 4% PFA. Parasagittal sections (12 μm thick) were stained with 0.1% cresyl violet/0.5% acetic acid. Images of whole sections were scanned using an NDP slide scanner (Hamamatsu Photonics). Quantitative analysis of the immunofluorescence signal was also performed on the 12-μm parasagittal or 10-μm coronal cryostat sections. Sections were postfixed with methanol for 10 min at −30 °C for GluA1 and synaptophysin staining; with the methanol/acetone treatment for PSD-95, GluN1 and GluN2A staining; or without pretreatment for GluA2 staining. After the sections were blocked with 2% normal goat serum, they were reacted with the primary antibodies at 4 °C overnight, followed by appropriate fluorescence-labelled secondary antibodies at room temperature for 1 h. The sections were transferred onto Superfrost slides (Matsunami Glass) and mounted under glass coverslips with Vectashield with 4′,6-diamidino-2-phenylindole (DAPI; Vector Laboratories). Fluorescence images of whole sections were scanned using a fluorescence microscope (BZ9000, Keyence) and hippocampus regions including CA1, CA3, and the dentate gyrus were scanned using a confocal microscope (Olympus FV1000) with a × 40 objective. For the puncta signal analysis for Vglut1, Vgat and GluN2A (Supplementary Fig. 4e–h), the fluorescence images were scanned using LSM800 (Zeiss) with × 63 objective. For comparisons between *Lrfn2* KO and WT, all stained images were taken with the same laser settings, and the fluorescence intensity was quantified using ImageJ software with the same parameters. DAPI counterstaining was used as a fluorescence control.

For Golgi staining, Brains of 8-week-old littermates were stained using modified Golgi-Cox impregnation of neurons performed with an FD Rapid GolgiStain kit (FD NeuroTechnologies). We prepared coronal sections (100 μm thickness) and examined the area of the CA1 stratum radiatum of the dorsal hippocampus. At least 11 pyramidal neurons per animal with clearly visible staining from the soma to the distal dendrites were randomly selected, and >2 dendritic segments per neuron that were located on secondary or tertiary distal dendrites were scanned (86 segments in 33 neurons from three KO mice, 62 segments in 22 neurons from two WT mice) using a brightfield microscope (Axioskop 2 plus, Zeiss) with a × 100 objective. Counting of the spines (protrusions) and morphometrical analysis of spines were performed as described^4^. Individual spines were manually traced using NeuroLucida software (MBF Bioscience), followed by measuring the maximum length and head width of each spine. The means of these parameters were calculated for each neuron and compared between genotypes.

For electron microscopic analysis, three anaesthetized littermate mice (8-week old) were perfused with 2.5% PFA/2% glutaraldehyde in 0.1 M sodium phosphate buffer (pH 7.4). Brains were sectioned on a Vibratome (VT1000 S, Leica) at 200 μm, osmicated with 1% OsO_4_ in phosphate buffer, dehydrated through a gradient series of ethanol, and then embedded in epoxy resin (EPSON812, TAAB). Ultra-thin sections of the hippocampus (70 nm) were cut with an ultramicrotome (EM UC6, Leica), collected on 200-mesh uncoated copper grids (G200HH, GILDER), and counterstained with uranyl acetate and lead citrate. The grids of the CA1 stratum radiatum (about 100 μm beneath the CA1 pyramidal cell body layer) were examined with a transmission electron microscope (JEM-1200EX, JEOL). Photographs were sequentially collected from proximal areas to distal areas using an FDL5000 imaging system (FUJIFILM). Asymmetric synapses that had clear synaptic structure consisting of a visible presynaptic membrane and visible postsynaptic membrane with single or multiple (perforated) PSDs were counted at × 10,000 original magnitude (13 grids from three *Lrfn2* KO and 11 grids from three WT). Mean synaptic densities were calculated for each grid and analysed for each genotype. For structural analysis, we obtained highly magnified photos (× 30,000) from the same grids. Every clear asymmetric synapse with a single PSD was manually analysed for the PSD length, PSD thickness and synaptic cleft width using ImageGauge software (FUJIFILM); in total, 301 synapses from 3 *Lrfn2* KO mice and 306 synapses from three WT mice were analysed. Statistical significance of the morphological data was determined using Student's *t*-test or a Kolmogorov–Smirnov test with a level of significance of *P*<0.05.

### Primary neuronal cultures and immunocytochemistry

Dissociated primary neuronal cultures were prepared from the hippocampi of E17–19 Sprague Dawley rats and from E18 to P0 mice. The hippocampi were dissociated with trypsin/EDTA solution. The clumps of cells were gently triturated and washed with fetal bovine serum, and the solution was replaced with Neurobasal medium supplemented with B-27 (Invitrogen) and 2 mM L-glutamine. The cells were plated onto poly-L-lysine–coated glass coverslips or culture dishes. Cultured cells were transfected with Lipofectamine 2000 (Invitrogen) in accordance with the manufacturer's protocol before miniature EPSCs were recorded, or with calcium phosphate precipitation for the biotinylation assay. For immunofluorescence staining of synaptic proteins, neurons were fixed with 4% PFA/4% sucrose in 0.1 M sodium phosphate buffer (pH 7.4) for 20 min on ice, followed by pre-chilled 100% methanol for 10 min at −20 °C. After the neurons were blocked with 5% normal goat serum, they were incubated with primary and fluorescently labelled secondary antibodies in PBS containing 1% normal goat serum. For NeuroLucida tracing, DIV10 neurons were fixed with the 4% PFA/4% sucrose solution and permeabilized with 0.2% Triton X-100 for 10 min. After incubation with an anti-MAP2 antibody in 1% normal goat serum, immunoreactive neurites were visualized using the avidin-biotin-complex method. A total of 8–12 neurons from at least three separate cultures were manually traced using NeuroLucida software; the total length of dendrites was measured, and the complexity of dendrite branching was analysed by Sholl analysis.

For the analysis human LRFN2 and its variants, hippocampal primary culture neurons were obtained from E17.5 rat embryos. Myc-Lrfn2 expression vectors were transfected at DIV7 using Lipofectamine 2000, and fixed at DIV11 (Fig. 9c right) or DIV30 (Fig. 9c left, quantitative analysis based on four independent experiments). Immunostaining was performed using anti-Myc rabbit antibody (Santa Cruz) and anti-PSD-95 antibody (NeuroMab) to detect Myc-Lrfn2 and PSD-95, respectively.

The cell surface biotinylation assay was performed with transfected rat hippocampal neurons (DIV14) as described previously^6^ with some modifications. The primary cultures were incubated with 0.25 μg ml^–1^ Sulfo-NHS-SS-biotin in PBS with 1 mM MgSO_4_ and 2.5 mM CaCl_2_ (PBS+) on ice for 20 min, then washed three times with cold PBS+ containing 50 mM glycine. Cell lysates were prepared using RIPA buffer, and cell surface biotinylated proteins were precipitated with Neutravidin-agarose and detected by immunoblot.

### Electrophysiology

For recording from hippocampal slices, hippocampal slices were prepared from 8- to 14-week-old *Lrfn2* KO mice and their littermates and allowed to recover for more than 2 h. For electrophysiological recordings, slices were transferred to a submersion-type recording chamber and perfused with an artificial cerebrospinal fluid (ACSF, 28 °C) that was bubbled with 95% O_2_ and 5% CO_2_ and contained 119 mM NaCl, 2.5 mM KCl, 4 mM CaCl_2_, 4 mM MgSO_4_, 1 mM NaH_2_PO_4_, 26.2 mM NaHCO_3_, 11 mM glucose and 0.1 mM picrotoxin. fEPSPs were recorded from the CA1 areas with a pipette filled with the ACSF using Multiclamp 700A (Molecular Devices). Schaffer collateral/commissural inputs were stimulated at 0.05 Hz with a glass pipette placed in the stratum radiatum. LTP was induced by two trains of 100 Hz 100 pulses at 20 s interval. To obtain paired-pulse ratio, two stimuli at intervals of 10, 20, 50, 100, 200, 500 and 1,000 ms were applied to the Schaffer collaterals. Post-tetanic potentiation was induced by 100 pulses at 100 Hz in the presence of D-APV (50 μM). We also performed voltage-clamp recordings using a Multiclamp 700A (Molecular Devices). Acquisition and analyses were performed using custom Igor Pro (WaveMetrics) software routines. Whole-cell recordings were made from principal neurons in the CA1 areas through recording electrodes (3–6 Mohm) filled with a solution containing 135 mM caesium methansulfonate, 8 mM NaCl, 10 mM HEPES, 0.2 mM EGTA, 4 mM MgATP and 0.3 mM Na_3_GTP (pH 7.2 with CsOH, osmolality adjusted to 275–285 mOsm). Series and input resistances were monitored online throughout the experiments. Initially, AMPAR-mediated EPSCs were obtained at −70 mV, then NMDAR-mediated EPSCs were recorded at +40 mV in the presence of CNQX (10 μM), and the ratio of the mean amplitude of these EPSPCs was defined as the AMPAR/NMDAR ratio. Miniature EPSCs (mEPSCs) were recorded at 4 KHz in the presence of TTX (1 μM) and analysed with Mini Analysis software (Synaptosoft). The threshold mEPSC amplitude was set at 5 pA and each mEPSC was verified visually. In silent synapse experiments, small AMPAR-EPSCs were evoked at −70 mV at 0.1 Hz through minimal stimulation at a reduced intensity at which some failures were surely identified visually, and then NMDAR-EPSCs were recorded using the same stimuli at +40 mV (refs 66, 67). Failure rates at −70 mV (*F*_–70_) and +40 mV (*F*_–70_) were estimated by doubling that of events with an amplitude of more than and less than zero, respectively^66^. The percentage of silent synapses was calculated using a formula 1−Ln(*F*_–70_)/Ln (*F*_+40_)^66^.

For the electrophysiological analysis using cultured hippocampal neurons, recording of mEPSCs was performed 7–10 days after transfection of a series of expression vectors, each combined with an EGFP vector. EGFP-positive pyramidal cells were identified under a inverted fluorescence microscope (IX71, Olympus) and examined using whole-cell patch-clamp mode. A patch-clamp amplifier (EPC-7, List Electronics, Darmstadt, Germany) was used for recording. Data were low-pass filtered at 3 kHz and digitally sampled at 10 kHz using pClamp8.0 (Molecular Devices, Union City, CA). The bath solution consisted of 137 mM NaCl, 4 mM KCl, 2 mM CaCl_2_, 1 mM MgCl_2_, 17 mM glucose and 10 mM HEPES-NaOH (pH 7.4). Tetrodotoxin (1 μM), picrotoxin (100 μM) and DL-AP5 (50 μM) were added during recording. The intrapipette solution consisted of 110 mM K-gluconate, 30 mM KCl, 2 mM MgCl_2_, 1 mM EGTA, 0.25 mM CaCl_2_, 3 mM Na_2_ATP, 0.3 mM GTP and 5 mM HEPES, pH 7.2. Patch electrodes had resistances of 3–5 Mohm. Neurons were voltage clamped at −70 mV. mEPSCs were detected and measured using the MINI ANALYSIS program (Jaejin Software, Leoniua, NJ).

### Subjects and mutation screening of LRFN2

We performed resequencing analysis of *LRFN2* in 165 patients with autism and 910 patients with schizophrenia who were of Japanese descent. For the Japanese sample, the diagnosis of schizophrenia was made based on DSM-IV by consensus of at least 2 experienced psychiatrists. The diagnosis of autism was made on the basis of the Autism Diagnostic Interview-Revised criteria^68^. All healthy control subjects were psychiatrically screened in unstructured interviews. All controls and patients gave informed, written consent to participate in the study after being provided with, and receiving an explanation of study protocols and objectives. This study was approved by the ethics committees of RIKEN and Hamamatsu University. We resequenced all the protein-coding exons and flanking introns. The detailed information is available on request. We tested common SNPs (minor allele frequency ≥5% in Japanese population; HapMap database (release 22), http://hapmap.ncbi.nlm.nih.gov) of *LRFN2* for genetic association with schizophrenia by studying two DNA samples: one includes 289 Chinese pedigrees with schizophrenia, consisting of nine trios and 279 quads, and the other includes 570 cases of schizophrenia and 570 controls from a Japanese population. The Chinese pedigree sample was made available by the NIMH Human Genetics Initiative, and detailed information about it can be obtained at http://nimhgenetics.org/.

### SNP selection and association study

We obtained a list of 52 tagged SNPs, which was expected to efficiently capture information of the common variations in and around the *LRFN2* (±10 kb) gene, by applying the Carlson's greedy algorithm^69^ to SNP genotype data of both Chinese and Japanese populations from the HapMap database (release 22). HapMap-LDSelect-Processor (http://bioapp.psych.uic.edu/HapMap-LDSelect-Processor.html) was used for this SNP tagging procedure, setting the *r*^*2*^ and the minor allele frequency threshold to 0.8 and 0.1, respectively. SNP genotyping was performed with the TaqMan system (Applied Biosystems, Foster City, CA) in accordance with the manufacturer's protocol. PCR was performed using an ABI 9700 thermocycler, and fluorescent signals were analysed using an ABI 7900 sequence detector single-point measurement and SDS v2.3 software (Applied Biosystems). The Haploview program^70^ was used for association testing. The transmission disequilibrium test and the allelic test were performed for the NIMH sample and the Japanese sample, respectively, to examine whether any alleles at *LRFN2* are transmitted significantly more often (over-transmitted) to offspring in the pedigrees or observed significantly more often (over-represented) in cases than expected by the null hypothesis of no association. *LRFN2* mRNA quantification was carried out using TaqMan probes and primers for *LRFN2* and *GAPDH* (an internal control) (TaqMan Gene Expression Assays gene expression products, Applied Biosystems).

### Statistical analysis

Data represent means±s.e.m. unless otherwise noted. Student's unpaired 2-tailed *t*-test or the Mann–Whitney *U*-test was used to determine the statistical significance of differences between two groups; single-factor ANOVA or repeated measures ANOVA was used to compare three or more groups, followed by the Tukey–Kramer post hoc test for multiple comparisons. Cumulative plots were analysed using the Kolmogorov–Smirnov test. Statistical analyses were performed in microsoft excel and SPSS (ver. 16.0, SPSS Japan).

### Data availability

All data generated or analysed during this study are included in this published article and its Supplementary Information files.

## Additional information

**How to cite this article:** Morimura, N. *et al*. Autism-like behaviours and enhanced memory formation and synaptic plasticity in Lrfn2/SALM1-deficient mice. *Nat. Commun.* **8,** 15800 doi: 10.1038/ncomms15800 (2017).

**Publisher's note:** Springer Nature remains neutral with regard to jurisdictional claims in published maps and institutional affiliations.

## Supplementary Material

Supplementary Information Supplementary Figures and Supplementary Tables.

Supplementary Movie 1 Wild-type mouse (black, resident) investigates the intruder mouse (brown).

Supplementary Movie 2 Lrfn2 KO mouse (black, resident) avoids the intruder mouse (brown) and hid itself under the bedding material.
