# Supplementary material for: Autism-like behaviours and enhanced memory formation and synaptic plasticity in Lrfn2/SALM1-deficient mice
Source: Nat Commun. 2017 Jun 12;8:15800. doi: 10.1038/ncomms15800 (PMC5472790; doi:10.1038/ncomms15800)
Supplement: Supplementary Information — Supplementary Figures and Supplementary Tables. [file ncomms15800-s1.pdf]

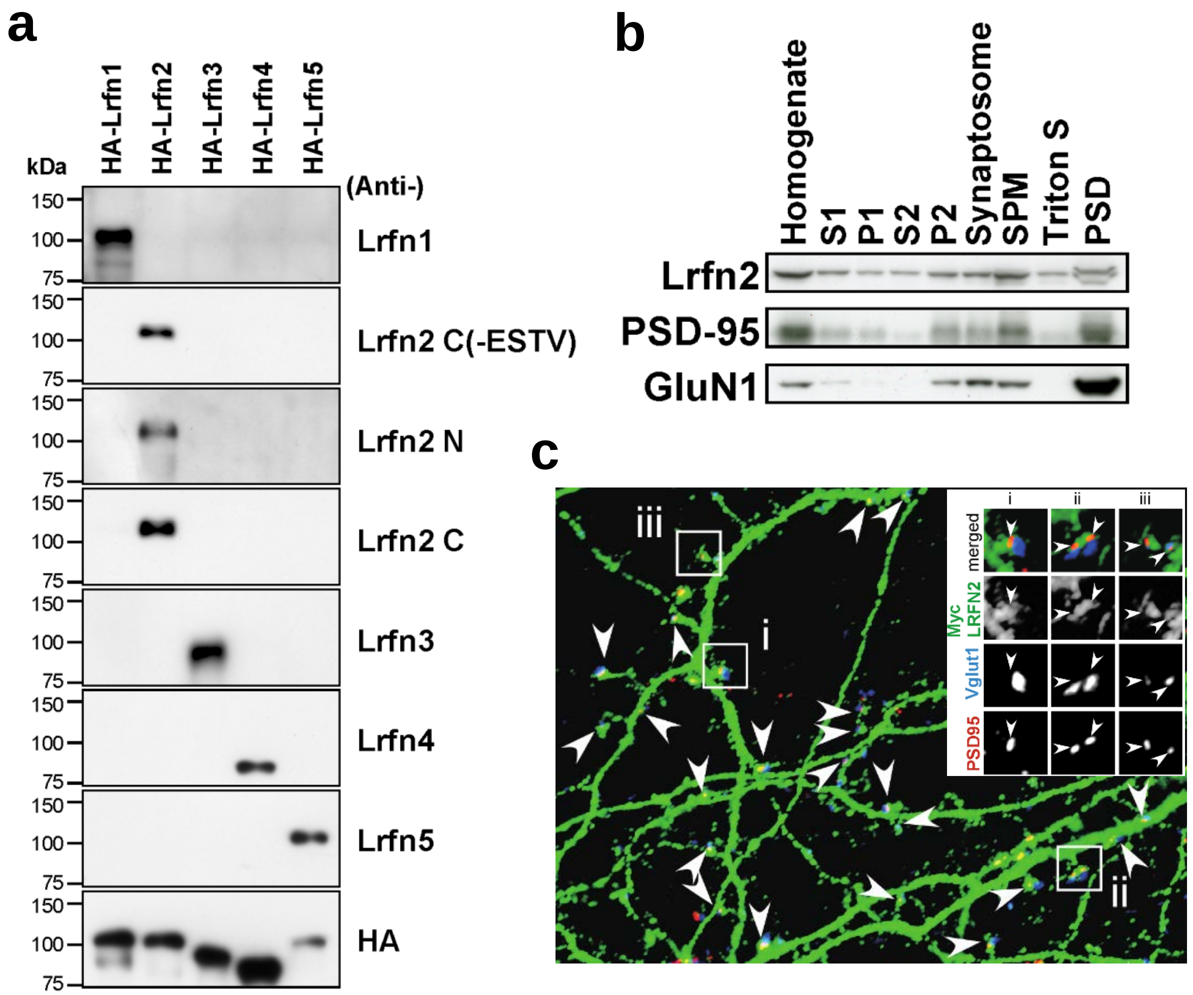

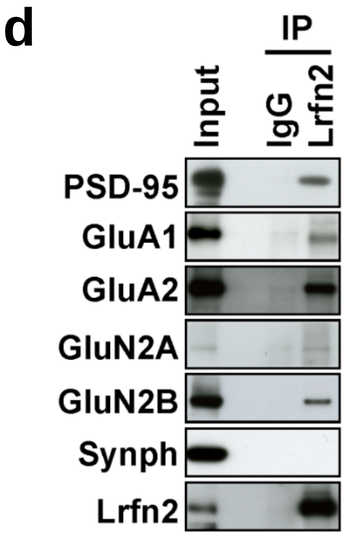

Supplementary Figure 1 related to Figure 1.
Specificity of antibodies and characterization of Lrfn2 in cultured hippocampal neurons.

(a) Full-length HA-tagged Lrfn1-5 were expressed into COS7 cells, and the cell lysates were probed with rabbit anti-Lrfns specific antibodies. The same lysates were also probed with an antibody of HA epitope tag that recognizes all HA-Lrfn proteins. Lrfn2N antibody reacts to the extracellular region, the others are against the intracellular regions. (b) Subcellular distribution of Lrfn2 protein. 4-week old mouse brains mainly containing hippocampus and cortex were homogenized and were detected by immunoblot analysis using antibodies of Lrfn2-N, PSD-95 and GluN1. S, supernatant; *P*, pellet; *SPM*, synaptosomal plasma membrane fraction; *Triton S*, supernatant after extraction of SPM with Triton X-100; *PSD*, postsynaptic density fraction. Homogenate sample was loaded with 25 µg protein per lane, and other fractions were loaded with 10 µg protein per lane. (c) Immunostaining of Myc-tagged human LRFN2 protein expressed in Lrfn2 KO hippocampal neurons. Antibodies against Myc (*MycLRFN2*, *green*), Vglut1 (*blue*), and PSD-95 (*red*) were used for the triple staining. *Arrowheads* indicate the $Myc^{+}PSD\text{-}95^{+}Vglut1^{+}$ synapses. *Inset* indicates each protein distribution in the three boxed regions (i – iii).

(d) Immunoprecipitation analysis of Lrfn2 protein. Mouse Lrfn2 proteins in synaptosomal fraction were precipitated with anti-Lrfn2C antibody or normal rabbit IgG as negative control. The synaptosomal fraction was prepared from adult mouse cerebral cortex and hippocampus. Immunoblotting revealed the immunocomplex of Lrfn2 contains PSD-95, GluA1, GluA2, GluN2A and GluN2B, but not synaptophysin. Input, 5%.

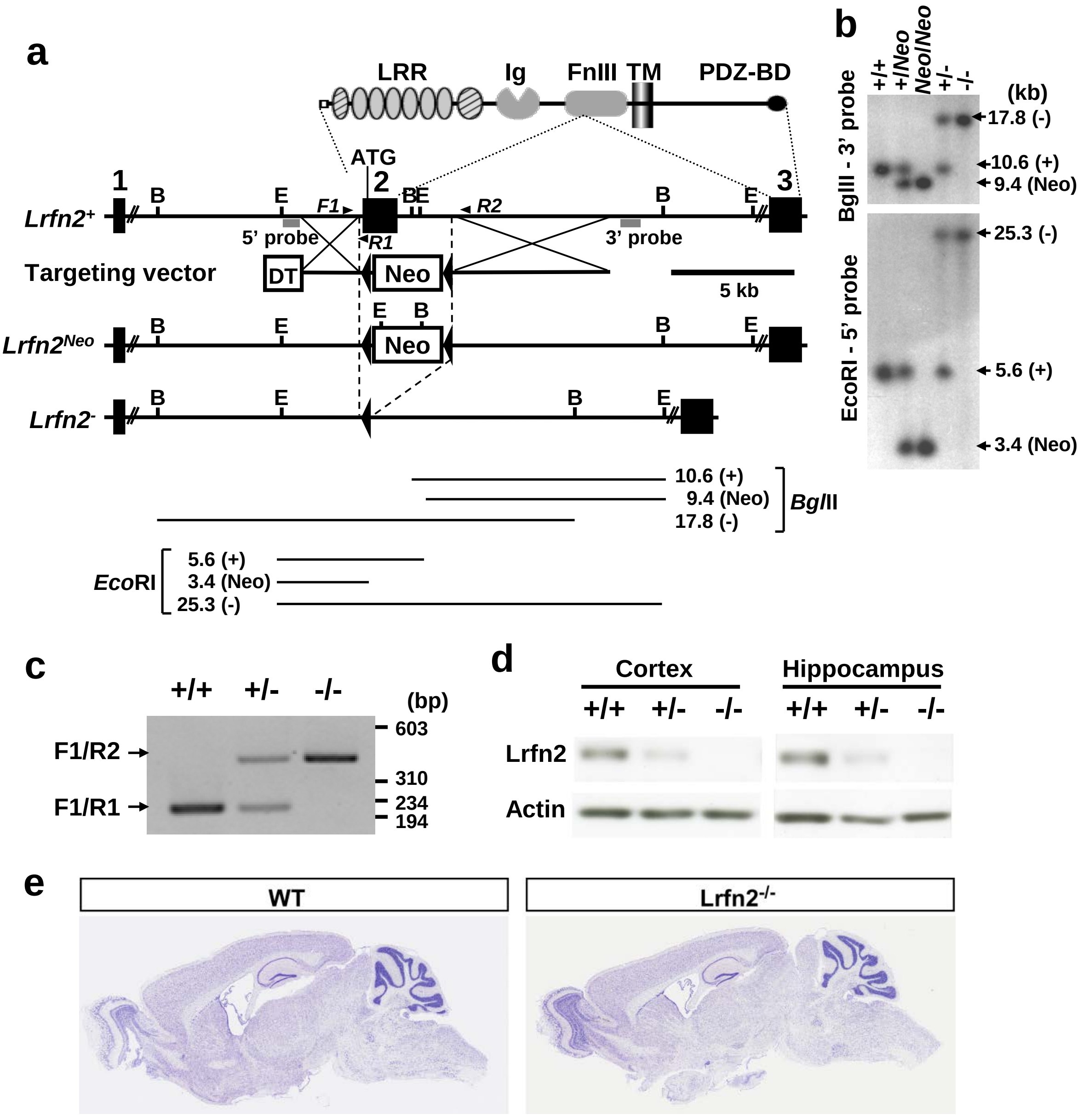

Supplementary Figure 2 related to Figure 2.
Generation and characterization of Lrfn2 KO mice.

(a) Targeting strategy for the disruption of the *Lrfn2* gene. Schematic diagram of domain structure of Lrfn2 protein, the mouse *Lrfn2* genomic structure with 3 exons are indicated by black boxes. The exon 2 including the translation initiation codon (*ATG*) was replaced by a floxed-neomycin resistance cassette. Homologous recombination results in an out-of-frame mutation in the *Lrfn2* gene. 5’- or 3’-probes are indicated by gray bar. Locations of PCR primers for genotyping are indicated by arrowheads (*F1*, *R1* and *R2*). Restriction enzyme site: *B*, BglII; *E*, EcoRI. *Black triangle*, loxP sequence; *Neo*, floxed-neomycin-resistant gene; *DT*, diphteria toxin. (b) Southern blot analysis of BglII-digested genomic DNA using 3’ probe (*upper panel*) or EcoRI-digested genomic DNA using 5’ probe (*lower panel*). (c) PCR genotype analysis of representative litter from heterozygote intercross. (d) Immunoblot analysis of Lrfn2 protein in the cortex and hippocampus from adult littermates. Anti-Lrfn2C antibody was used for the detection. (e) Normal histology in cresyl violet staining of adult *Lrfn2* KO brain.

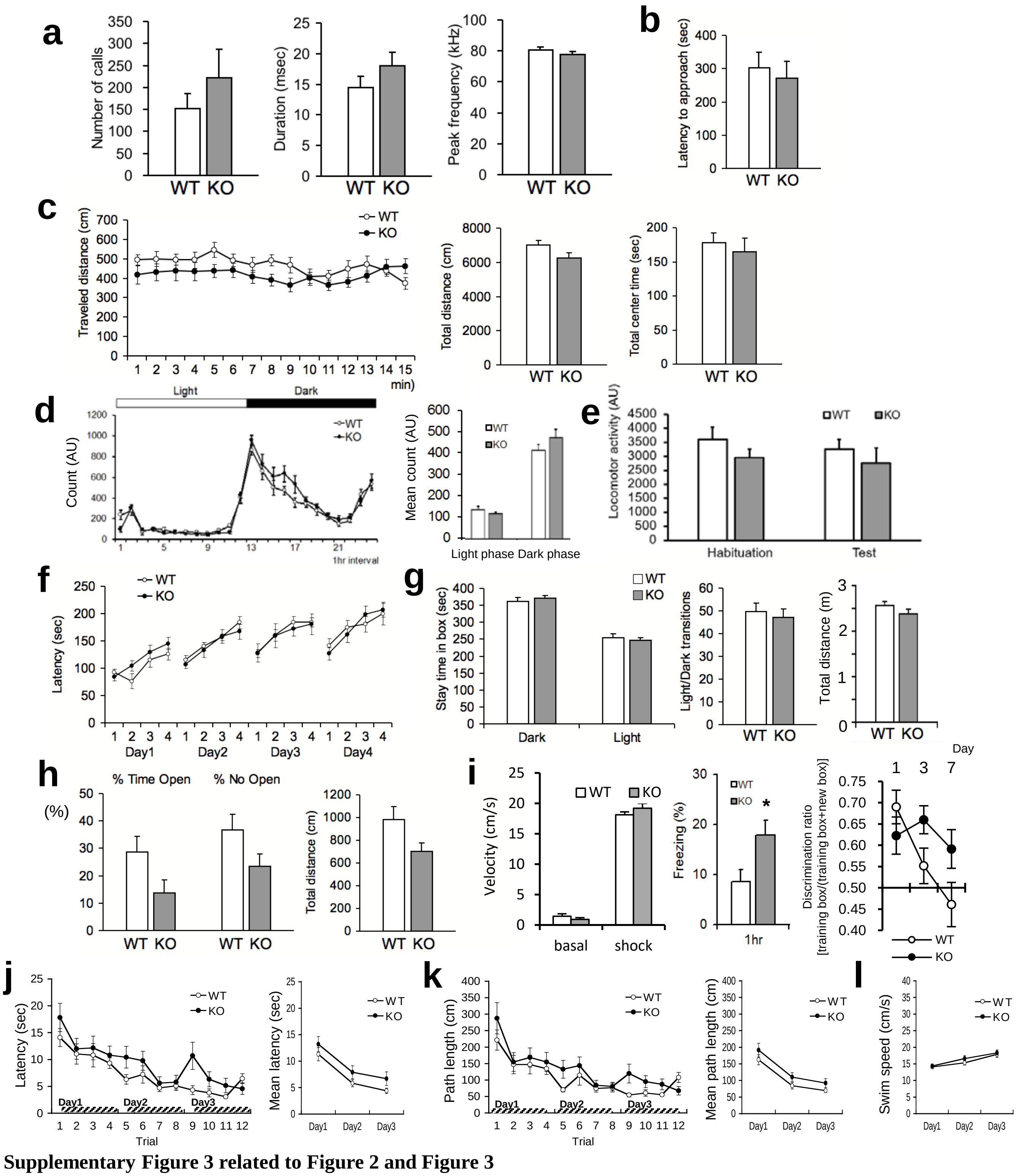

Supplementary Figure 3 related to Figure 2 and Figure 3
Supplemental results for Lrfn2 KO behavioral tests.

(a) Isolation induced USV emission. USV emissions were recorded from the maternally isolated mice at 7 days after birth. WT, n = 11 mice; KO, n = 10 mice. Number, duration, and peak pitch of the USV elements were not significantly different between the two genotypes. (b) Hidden-cookie finding test of olfaction. (c) Open field test. (*left*) Time course of locomoter activity indicated by traveled distance in the open arena. (*middle*) Total distance traveled during the 15 min test period. (*right*) Total center time during the test period. (d) Spontaneous motor activity in homecages (mean of 7 days recording). (e) Locomotor activity during marble burying test. (f) Accelerating rotarod test. Latency times to fall from the rotating rod are indicated. (g) Light/dark transition test. (left) Time stayed in dark or light box. (middle) Number of light-dark boxes transition. (*right*) Total distance traveled. (h) Elevated plus maze test. For all tests in (b-h), WT, n = 10 mice; KO, n = 10 mice. (i) Fear conditioning. (left) Movement velocity before (basal) and after footshock. (*middle*) Freezing 1 h after conditioning. $*P < 0.05$ (*right*) Discrimination ratio. $P = 0.051$ (Day 3), $P = 0.068$ (Day 7) in *t*-test. $P = 0.050$ in two-way repeated measures ANOVA (genotype×day). (j-l) Morris water maze test using a visible platform. The latency to reach the platform (j), path length (k), and swim speed (l) in *Lrfn2* KO mice were comparable to those in WT littermates, indicating that visual recognition, motor skills, and motivation are not impaired in *Lrfn2* KO mice.

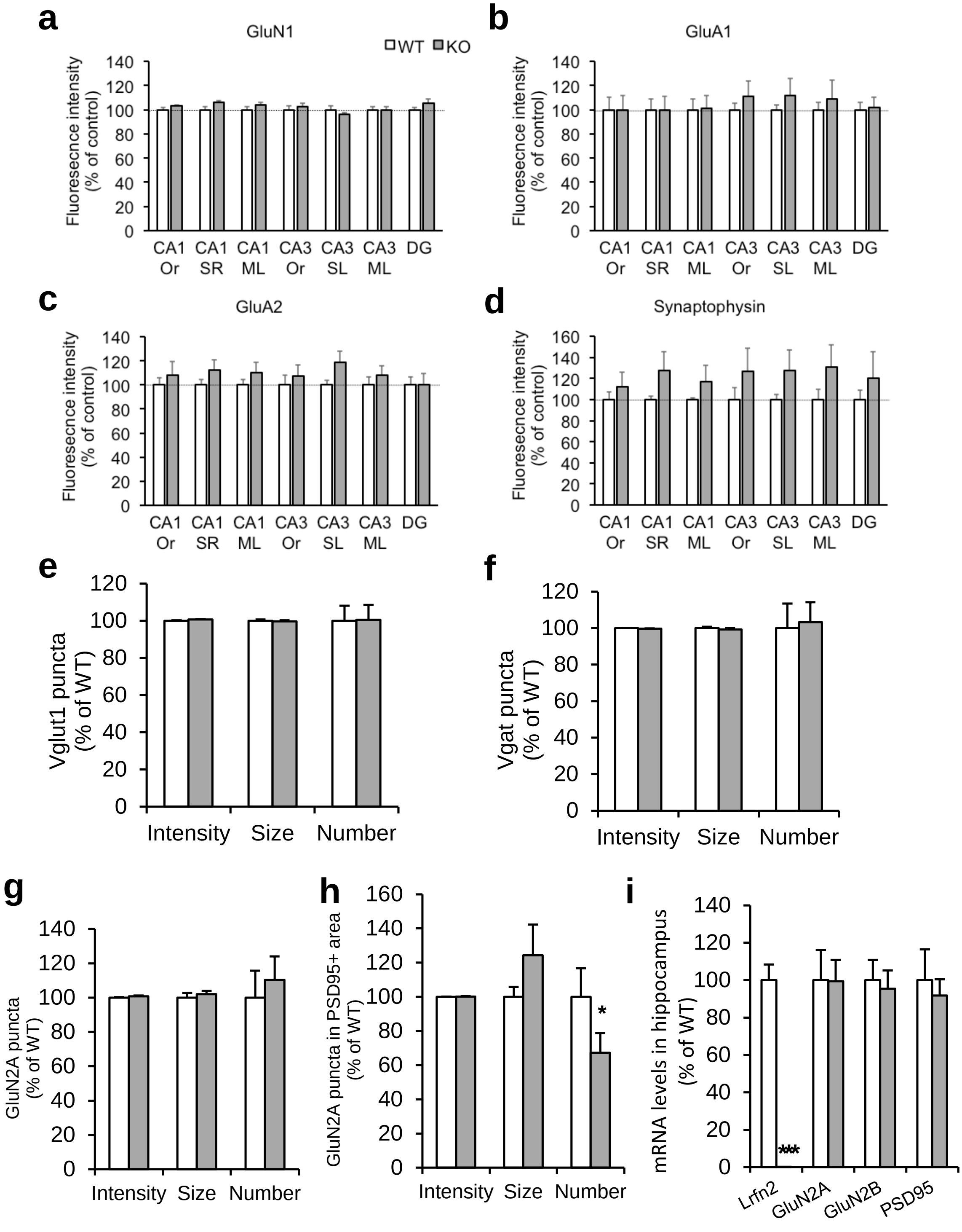

Supplementary Figure 4 related to Figure 4.
Quantification of additional synaptic proteins in *Lrfn2* KO hippocampus by immunostaining.

(a-d) Immunofluorescence staining was carried out in 8-week-old WT and KO hippocampus, and fluorescent intensities of CA1, CA3 and DG were quantified for Synaptophysin, GluA1, GluA2 and GluN1. Or, oriens layer; Rad, radial layer; Lmol, lacunosum molecular layer; Slu, stratum lucidum layer; Mol, molecular layer. n = 6–8 sections from 3–4 mice per genotype. (e-h) Immunostaining was carried out in 5-8 months-old male WT and KO hippocampus. Intensity, size, and number of Vglut1 puncta (e), Vgat puncta (f), GluN2A puncta (g), and GluN2A puncta in PSD95-positive area (h) were quantified in CA1 radial layer. n = 24-46 images from 5-6 WT mice; n = 28-47 images from 6-7 KO mice. (i) Quantitative PCR analysis of mRNA levels. Values were normalized by those of GAPDH mRNA. WT, n = 6 hippocampi from 3 mice; KO, n = 10 hippocampi from 5 mice. (a-i) open bar, WT; gray bar, KO. *$P < 0.05$, *** $P < 0.001$, *t*-test.

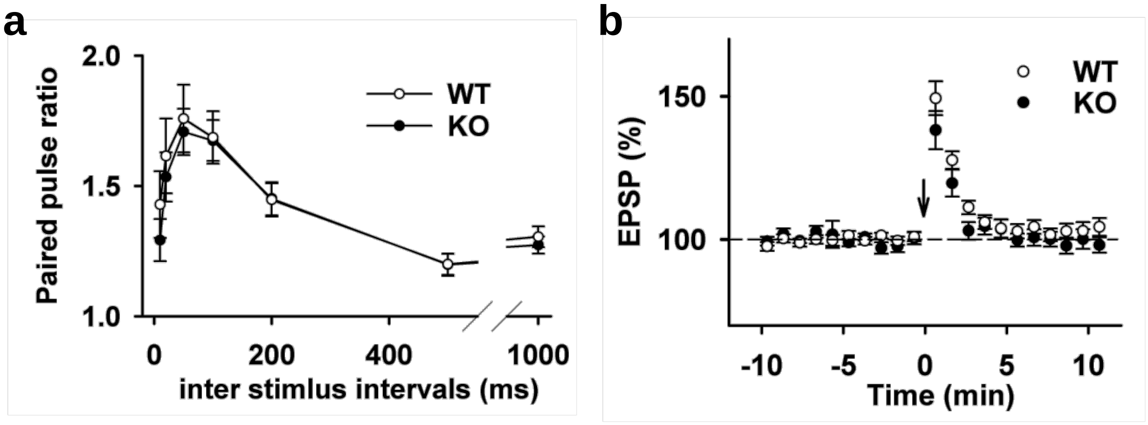

Supplementary Figure 5 related to Figure 6. Paired-pulse ratio and posttetanic potentiation in *Lrfn2* KO hippocampus CA1 region.

(a) Summary of paired-pulse ratio (n = 8 slices from 4 WT, n = 10 slices from 5 KO mice). The loss of Lrfn2 function had no effect on the release probabilities. (b) Summary of posttetanic potentiation (WT, n = 13 slices from 3 mice; KO, n = 11 slices from 3 mice).

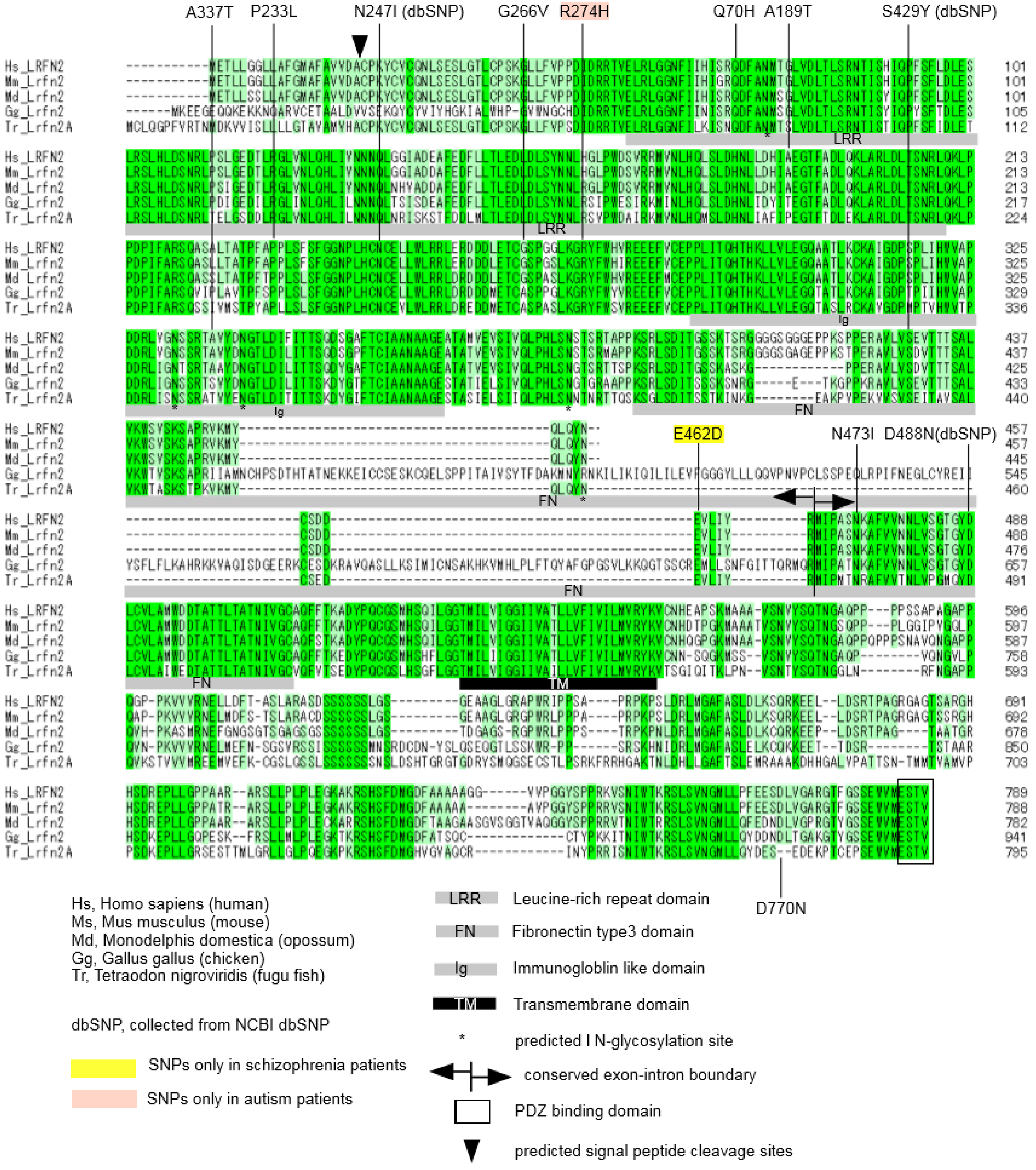

Supplementary Figure 6 related to Figure 9.
Location of the nonsynonymous SNPs of LRFN2 shown with multiple alignments of five vertebrate species.

Hs, *Homo sapiens* (human); Mm, *Mus musculus* (mouse); Md, *Monodelphilus domestica* (oppossum); Gg, *Gallus gallus* (chicken); Tr, *Tetraodon nigroviridis* (fugu fish). *Dark green*, absolutely conserved residues among the five orthologs; *light green* conserved in more than half orthologs.

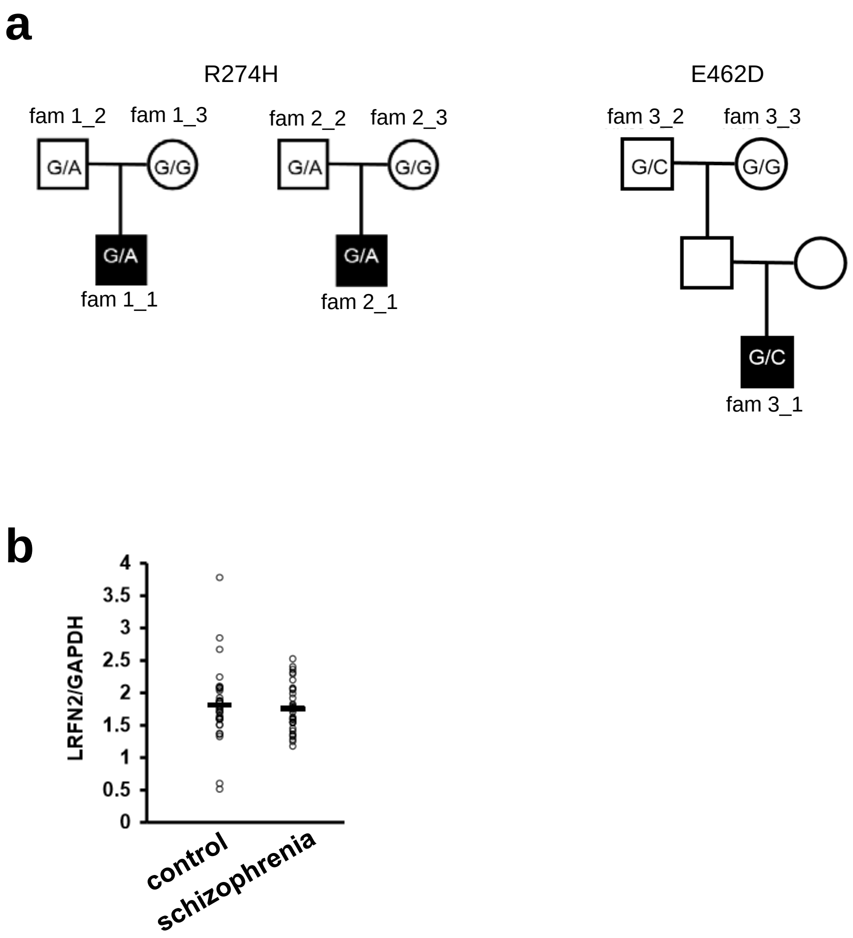

Supplementary Figure 7 related to Figure 9.
Supplemental results for resequencing analysis of human LRFN2 in neuropsychiatric disorder patients.

(a) Family-based association analysis of R274H and E462D. (b) Quantitative RT-PCR in postmortem brains. *LRFN2* mRNA amounts in postmortem brains from schizophrenia and control subjects. RNA from the dorsolateral prefrontal cortex (Brodmann’s area 46) from 35 schizophrenia patients and 35 controls were subjected for quantitative RT-PCR analysis. There was no significant difference between the two groups by *U*-test.

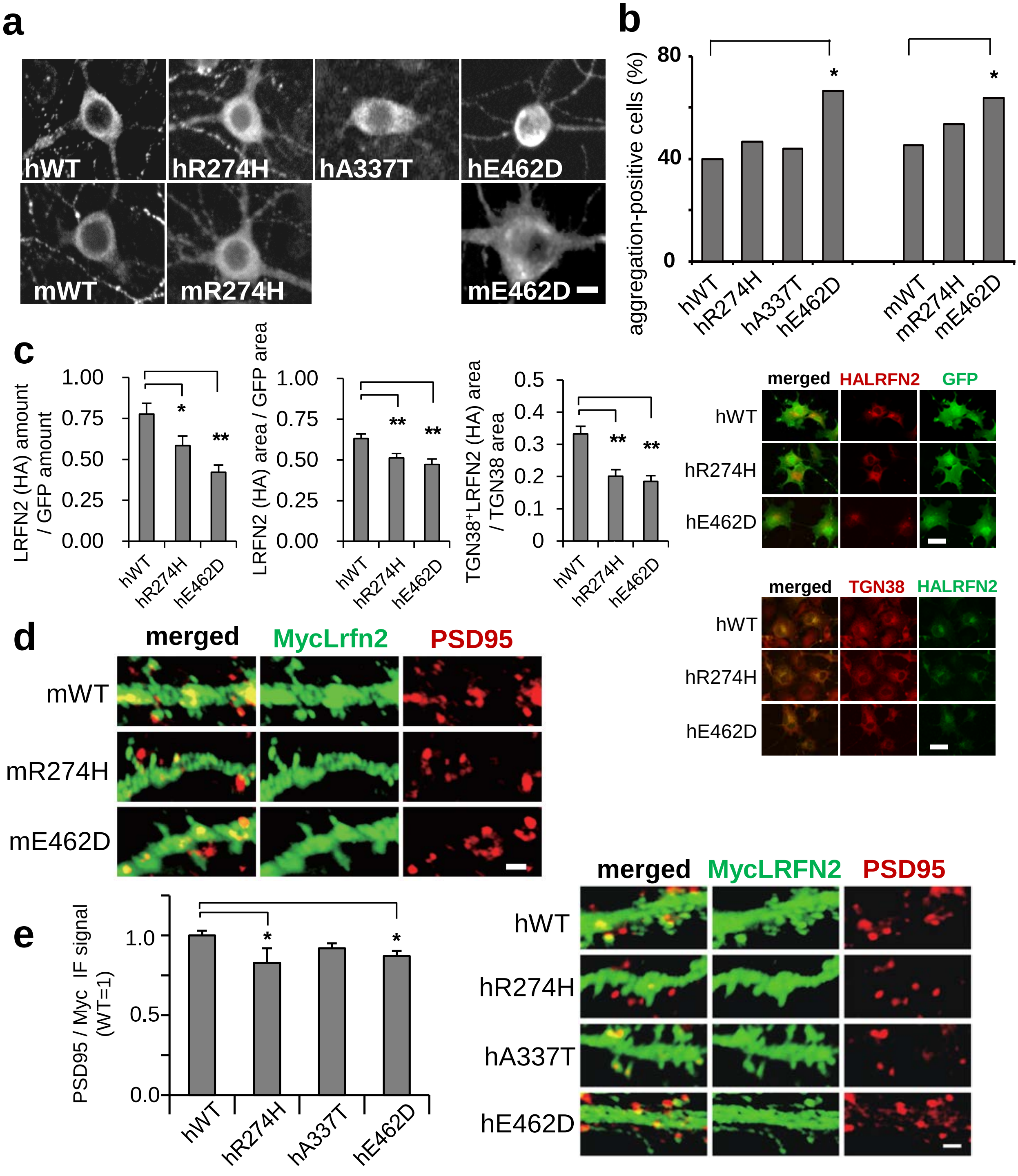

Supplementary Figure 8 related to Figure 9.
Supplemental results for resequencing analysis of human LRFN2 in neuropsychiatric disorder patients.

(a) Representative images for the localization of WT or mutant Lrfn2/LRFN2 proteins in the cell bodies of transfected hippocampal neurons (*scale bar*, 10 μm). (b) Percentages of the aggregate-containing cell bodies among the transfectants. (n = 75-88 cells from 15 images for each group; $*P < 0.05$, compared to WT chi square-test). (c) R274H and E462D cause protein amount and trafficking abnormalities. COS7 cells were transfected with HA-LRFN2 WT, R274H, or E462D in pCAG-ires-GFP vector. Protein localization was examined by immunostaining using antibodies against HA epitope, TGN38 (trans golgi network marker), or GFP. (*left*) For each experimental group, n = 55-59 (GFP), n = 27-29 (TGN38) images each containing at least 3 cells; $*P < 0.05$, $**P < 0.01$, compared to WT, *t*-test; (*right*) Representative images. *Scale bars*, 50 μm. (d) Representative images showing PSD-95 signals overlapping with transfected mouse Lrfn2 (m) proteins (*scale bar*, 5 μm). Similar results were obtained in 3-6 independent experiments (b, c). (e) Effects of the identified mutations on PSD-95 signals overlapping with transfected human LRFN2 proteins in Lrfn2 KO hippocampal neurons (*left*). Values for WT were adjusted to 1. n = 5-15 images for each group; $*P < 0.05$, compared to WT, *t*-test. Representative images (*right*; *scale bar*, 5 μm).

Supplementary Figure 9-1   Full blot

Full blots for Figure 1a

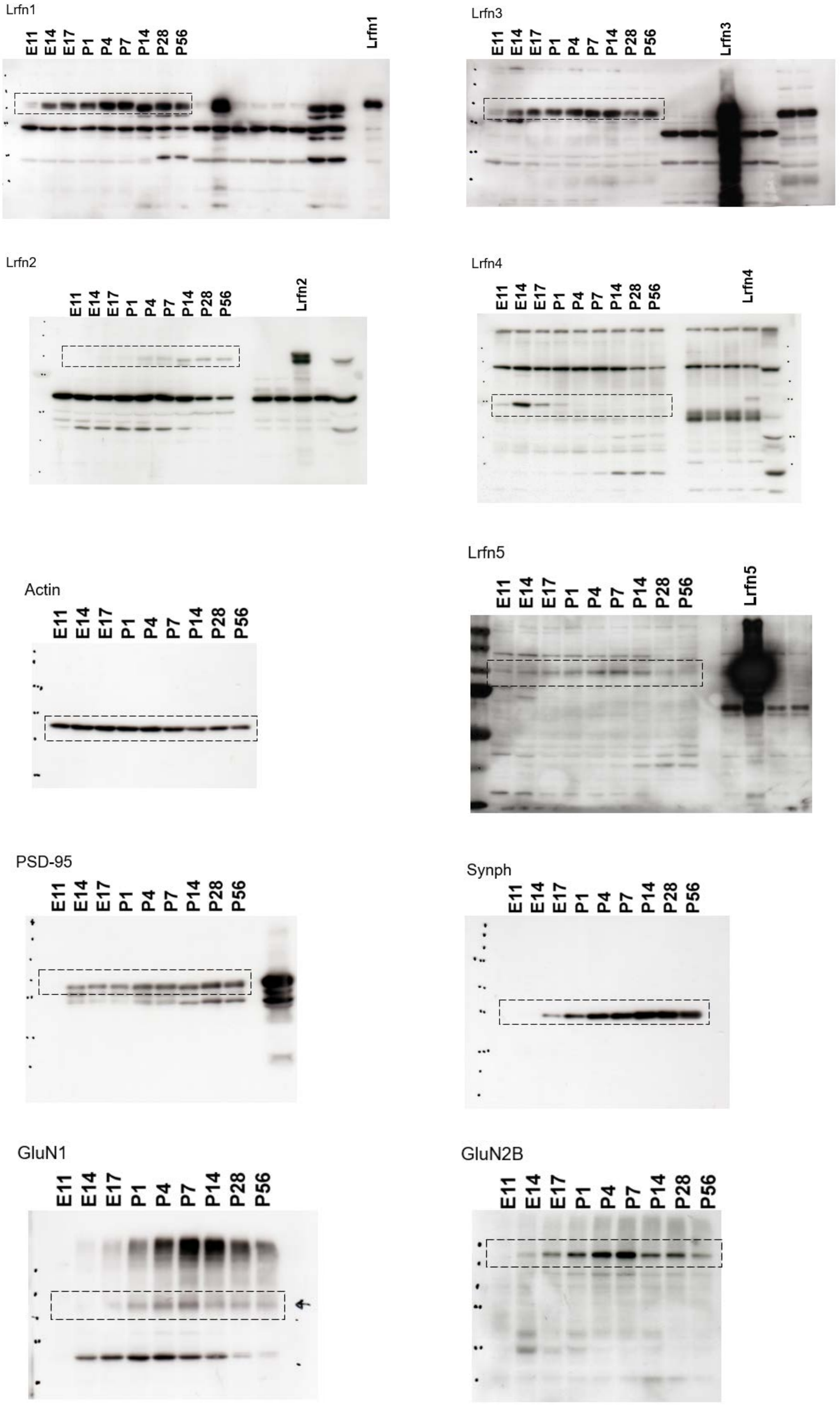

Supplementary Figure 9-2 Full blot

Full blots for Figure 1b

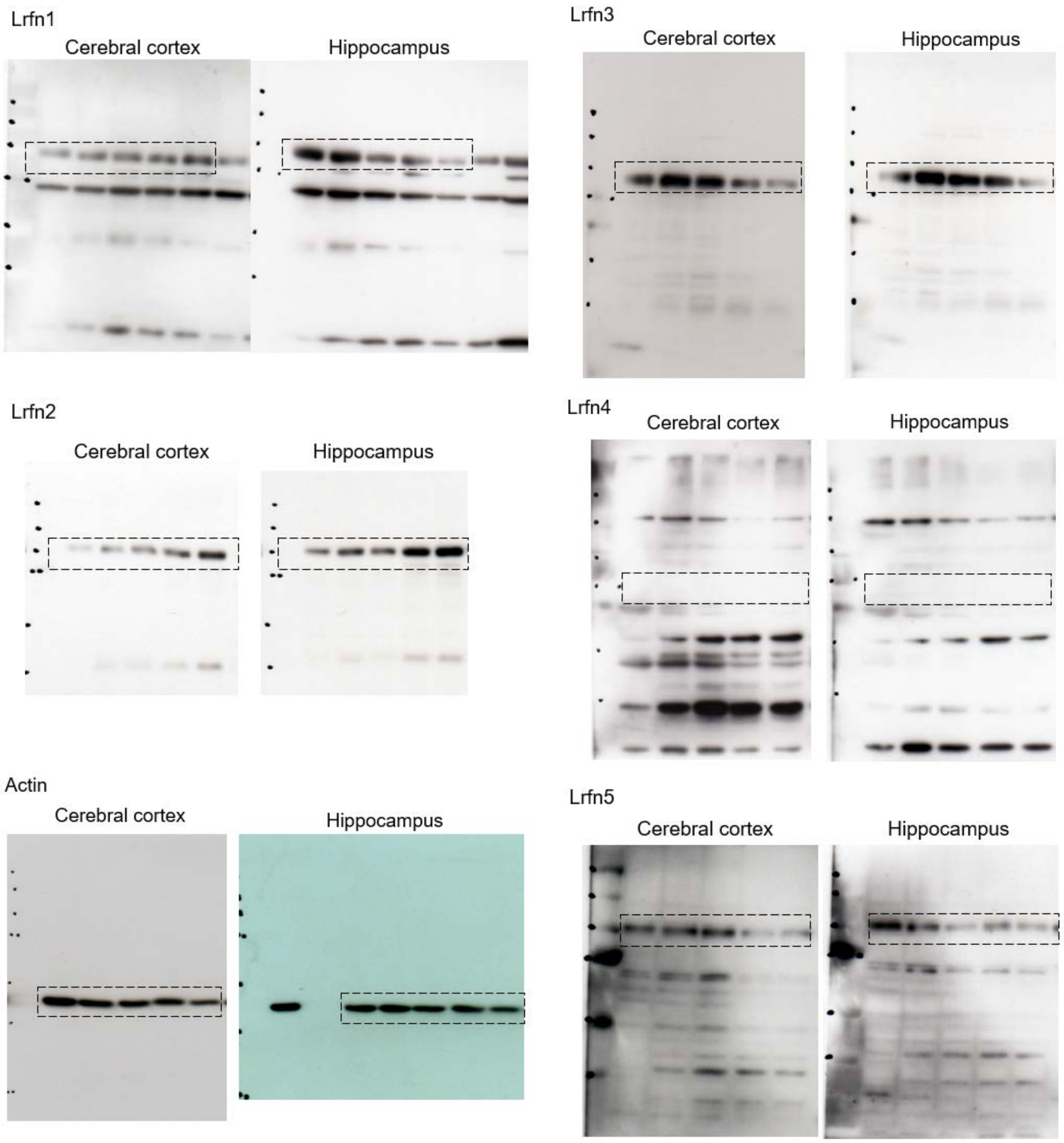

Full blots for Figure 1c

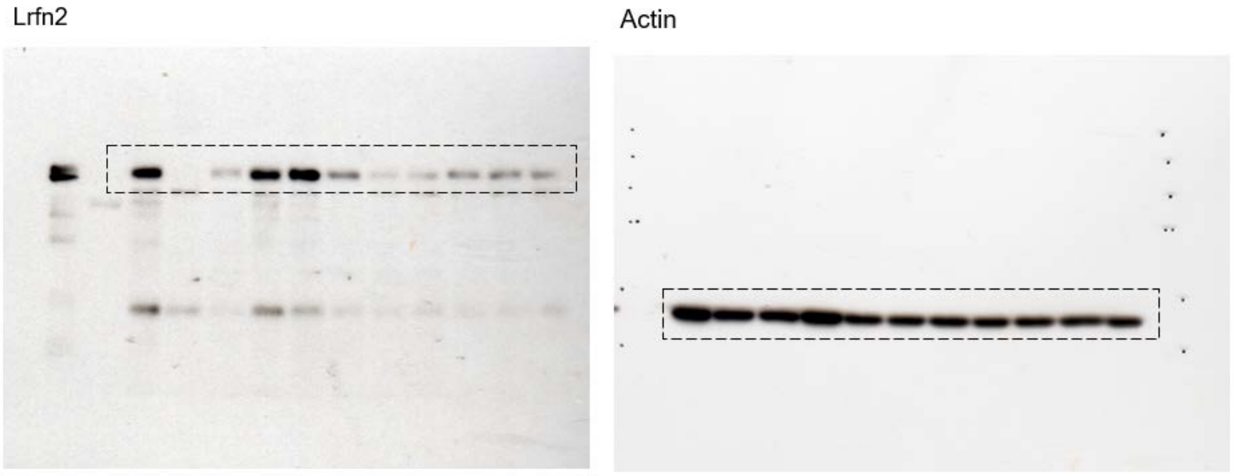

Supplementary Figure 9-3 Full blot

Full blots for Figure 7

GluN1

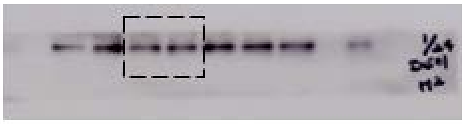

GluA1

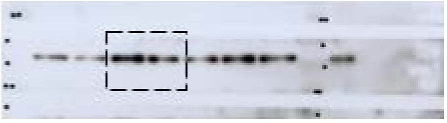

GluA2

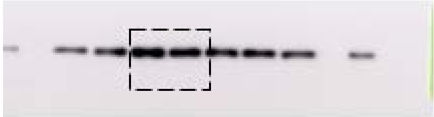

Actin

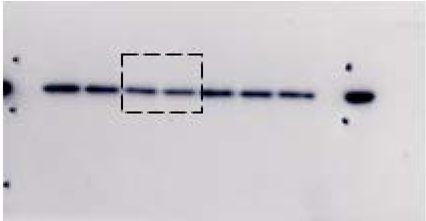

Full blots for Figure 8a

Myc

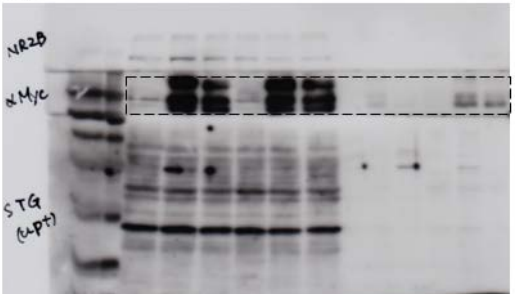

Flag

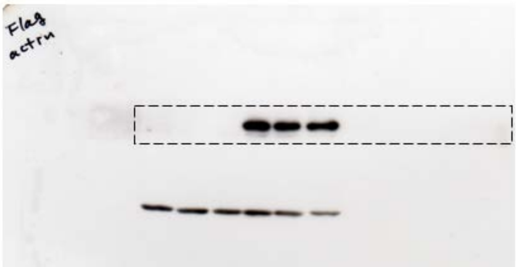

GluA1

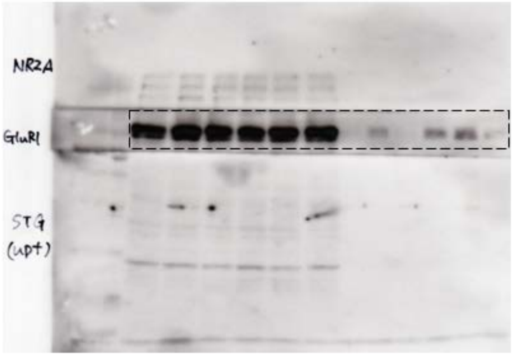

GluA2

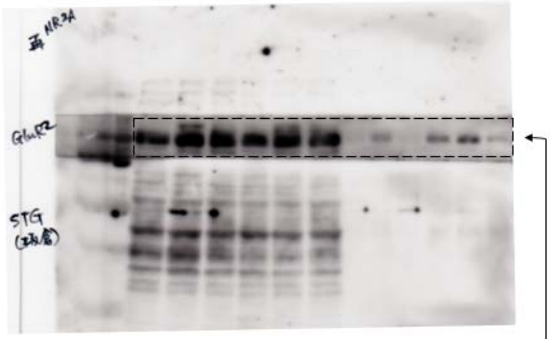

GluN1

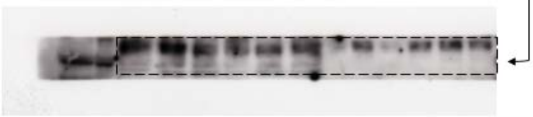

Tubulin

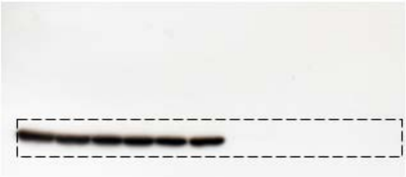

Supplementary Figure 9-4   Full blot

Full blots for Figure 9d

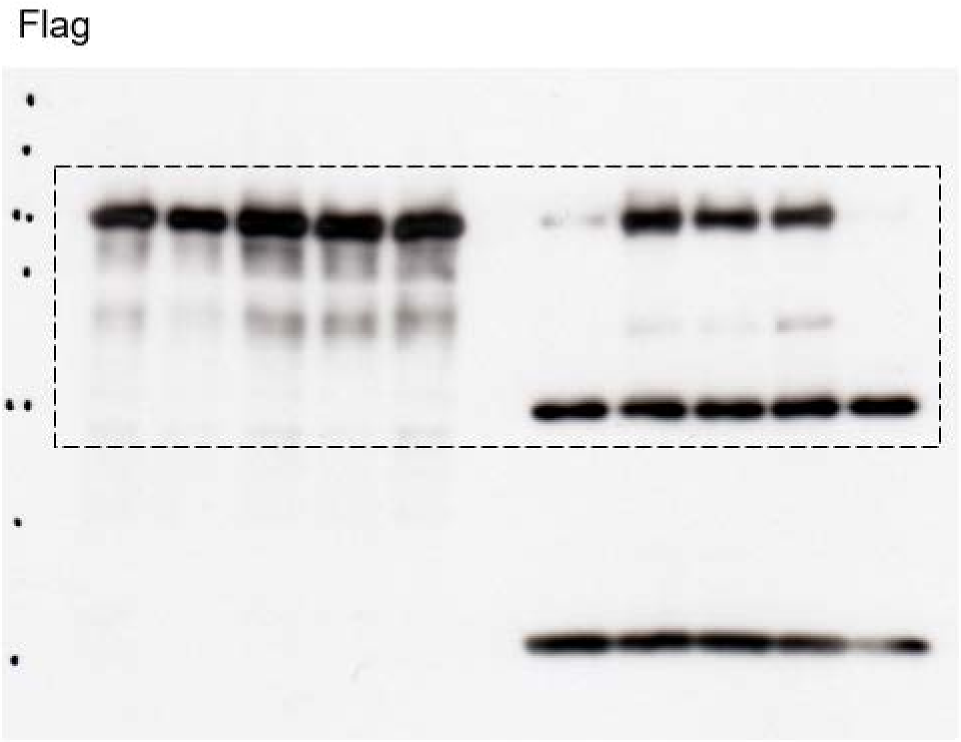

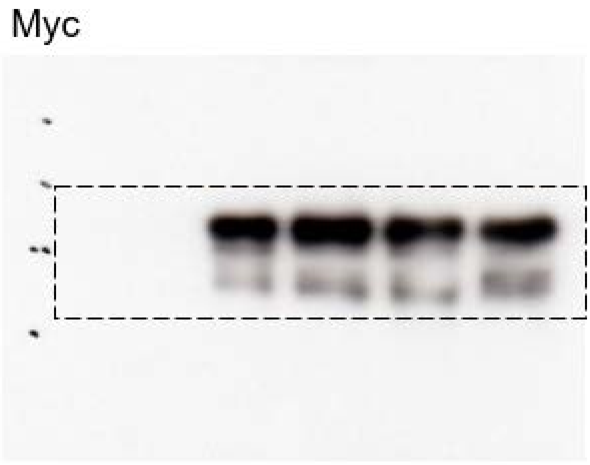

Supplementary Figure 9-5 Full blot

Full blots for
Supplementary Figure 1a

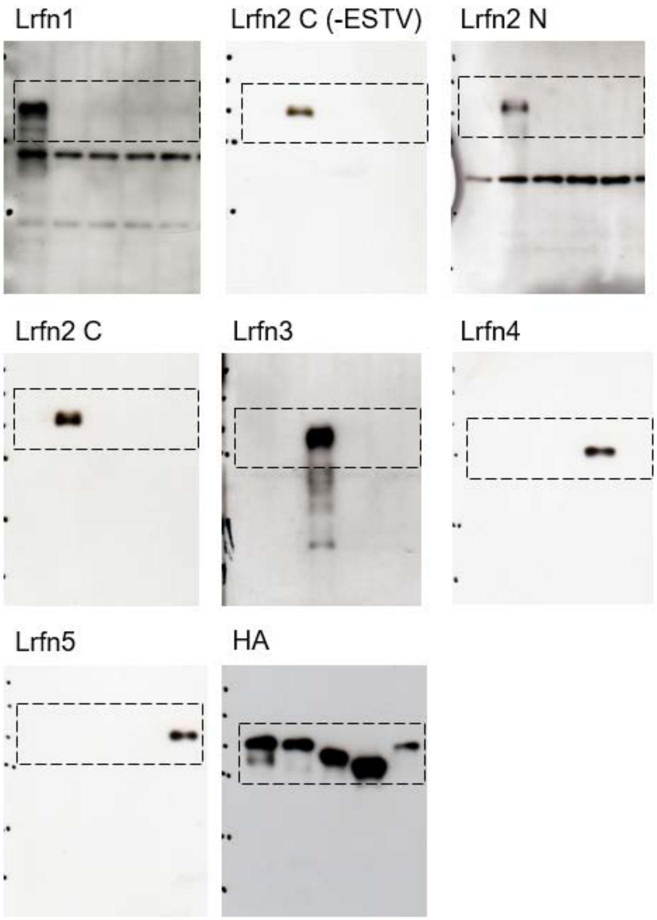

Full blots for
Supplementary Figure 1b

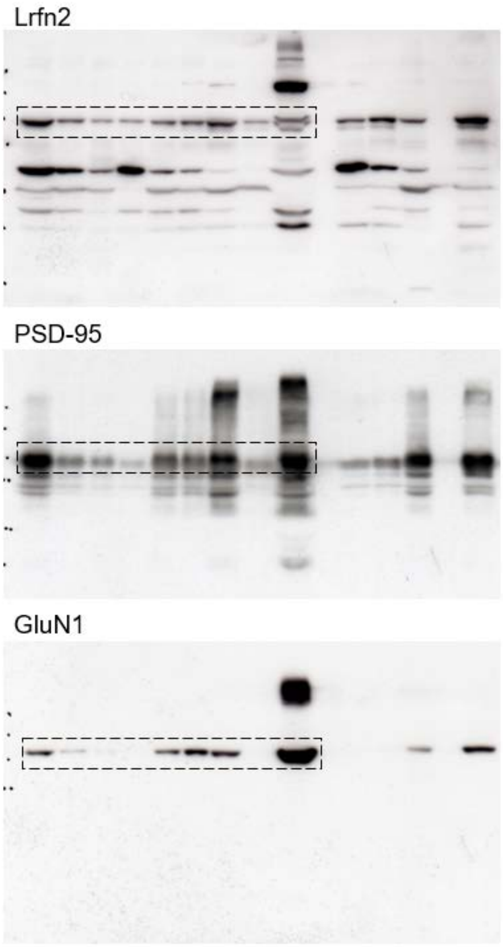

Supplementary Figure 9-6 Full blot

Full blots for Supplementary Figure 1d

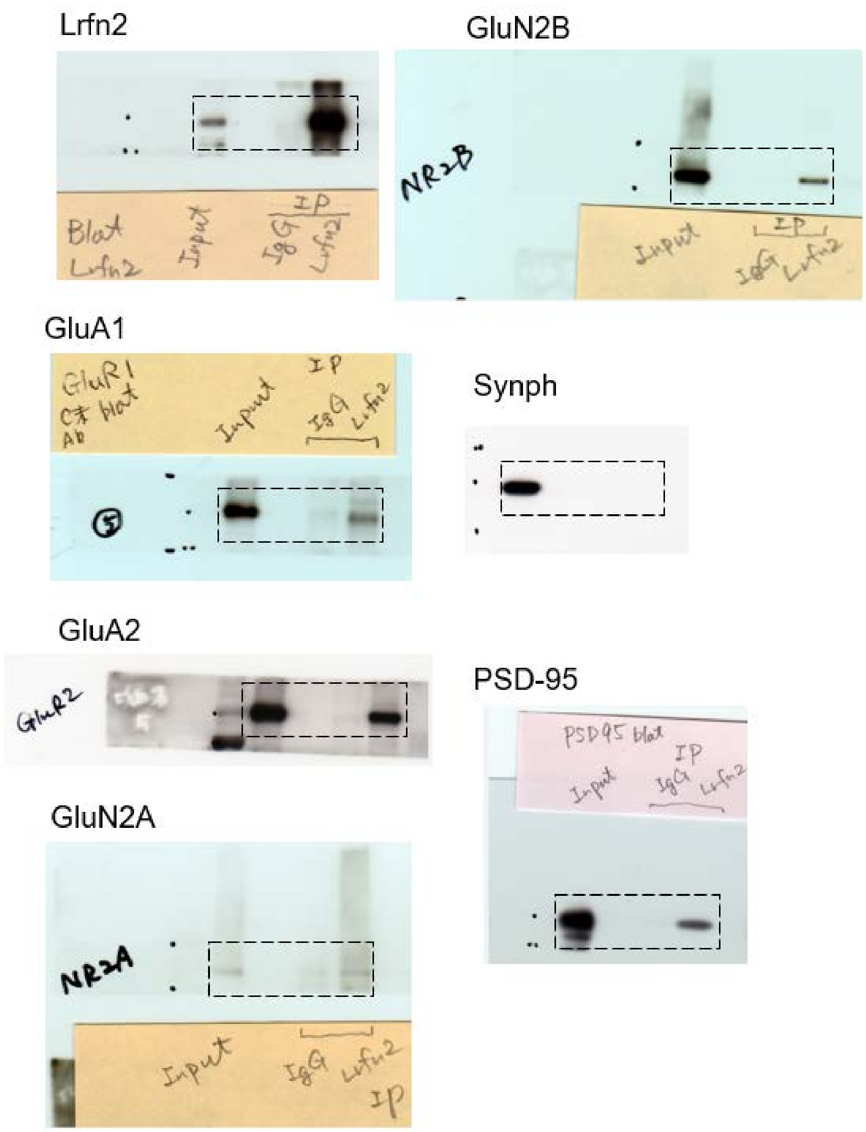

Full blots for Supplementary Figure 2d

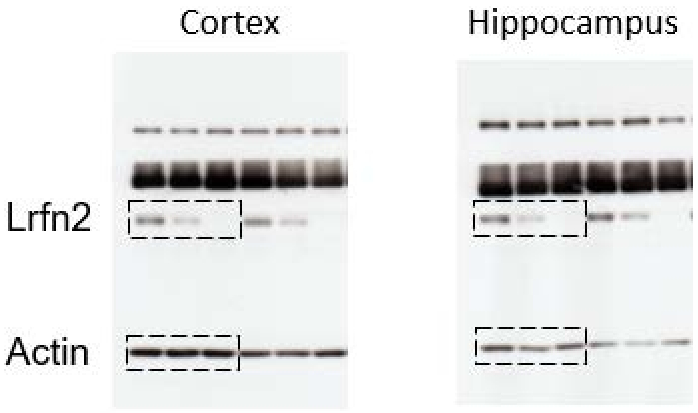

Supplementary Figure 9. Full blot pictures.
Untrimmed immunoblot pictures for Figures 1a-c, 7, 8a, and 9d, and Supplementary Figure 1a, 1b, 1d , and 2d.

Supplementary Table 1. Expression levels of synaptic proteins in Triton-X100 insoluble fraction from adult hippocampus

Synaptic proteins	Protein levels in Triton X-100 insoluble fraction (% ± SEM)		P-value
	WT (n=8)	KO (n=9)	
GluA1	100.0 ± 9.3	68.5 ± 8.2 *	0.016
GluA2	100.0 ± 12.4	85.2 ± 10.7	0.348
GluN1	100.0 ± 8.7	102.1 ± 13.4	0.891
GluN2A	100.0 ± 13.6	111.6 ± 22.5	0.658
GluN2B	100.0 ± 18.0	90.1 ± 18.3	0.688
GABAR [1)]	100.0 ± 14.0	118.5 ± 24.4	0.443
Synapsin I [1)]	100.0 ± 7.4	107.8 ± 12.3	0.562

1) WT, n=5; KO, n=5

* $p<0.05$

Supplementary Table 2. Linkage analysis of tag SNPs in LRFN2 regions with Asian schizophrenia and autism patients

			NIMH Chinese pedigrees								Japanese cases and controls							
SNP ID	Position[a]	Alleles	Minor allele	Frequency	Over-transmitted	Transmitted	Untransmitted	Chi square	P value	HWpval[b]	Minor allele	Frequency	Over-represented	case ratio[c]	control ratio[c]	Chi square	P value	HWpval[b]
rs1433723	40,458,377	AG	G	0.155	G	150	128	1.741	0.187	0.2266	G	0.169	A	939:187	943:197	0.182	0.6693	0.8158
rs16863	40,458,913	GA	G	0.399	G	249	239	0.205	0.6508	0.0612	A	0.394	A	448:674	443:695	0.237	0.6264	0.3842
rs2916256	40,459,030	AG	G	0.297	A	229	206	1.216	0.2701	0.072	G	0.22	A	892:236	873:263	1.637	0.2007	0.2547
rs6458154	40,461,984	CT	T	0.458	T	269	267	0.007	0.9312	0.3237	T	0.391	C	698:424	676:458	1.599	0.206	0.1271
rs6903068	40,462,294	CA	A	0.307	-	238	238	0	1	0.8082	A	0.176	A	215:911	182:952	3.617	0.0572	0.8003
rs1433721	40,462,772	CG	C	0.321	C	228	225	0.02	0.8879	0.0474	G	0.484	C	574:524	579:557	0.383	0.5361	0.7269
rs2436728	40,473,579	TC	C	0.497	C	280	246	2.198	0.1382	0.1891	C	0.412	C	475:649	456:680	1.047	0.3062	1
rs17681686	40,475,116	CG	G	0.179	C	175	156	1.091	0.2963	0.9492	G	0.363	G	410:718	410:724	0.009	0.9242	0.3041
rs934465	40,478,477	TC	C	0.492	T	264	233	1.934	0.1644	0.008	C	0.371	C	419:709	422:716	0.001	0.9753	0.0848
rs13207019	40,483,083	GA	A	0.319	A	242	235	0.103	0.7486	0.5565	A	0.464	G	611:515	599:531	0.357	0.5504	0.33
rs752556	40,485,971	TA	A	0.299	A	255	205	5.435	0.0197[d]	0.7769	A	0.273	T	819:305	823:313	0.05	0.8238	0.0979
rs2033528	40,487,877	CT	T	0.321	T	257	208	5.163	0.0231[d]	0.2204	T	0.323	T	366:762	364:770	0.031	0.8595	0.1823
rs13205455	40,494,953	CT	T	0.32	T	240	226	0.421	0.5166	0.4455	T	0.431	C	649:477	638:498	0.502	0.4786	0.5044
rs9394689	40,496,545	TC	C	0.182	C	177	154	1.598	0.2062	1	C	0.15	C	180:948	160:976	1.556	0.2123	0.3457
rs13196792	40,500,347	GA	A	0.292	G	226	221	0.056	0.8131	0.4349	A	0.478	G	597:529	581:551	0.65	0.4203	0.3017
rs6931355	40,502,453	TG	G	0.312	G	246	207	3.358	0.0669	0.1373	G	0.26	G	301:825	284:836	0.551	0.458	0.4007
rs4714347	40,514,901	TC	C	0.203	C	189	160	2.41	0.1206	0.325	C	0.227	T	871:255	868:256	0.005	0.9417	0.9472
rs6910503	40,515,958	GA	A	0.15	A	160	125	4.298	0.0382[d]	0.8367	A	0.158	A	179:947	179:955	0.005	0.9418	1
rs919875	40,519,678	TC	C	0.144	C	137	134	0.033	0.8554	1	C	0.069	C	78:1048	78:1060	0.005	0.9453	0.314
rs4236068	40,521,011	AG	G	0.083	A	91	78	1	0.3173	1	G	0.086	A	1031:91	1029:103	0.7	0.4028	1
rs10947890	40,523,715	GC	C	0.294	C	235	215	0.889	0.3458	1	C	0.238	G	866:260	856:278	0.632	0.4266	0.2202
rs12198137	40,523,897	GT	T	0.347	G	261	249	0.282	0.5952	1	T	0.333	G	752:370	751:379	0.08	0.7768	0.5849
rs1433734	40,524,778	GT	T	0.123	T	125	115	0.417	0.5186	0.988	T	0.158	G	959:167	943:189	1.478	0.2241	0.8965
rs1465718	40,528,938	CG	G	0.181	G	177	146	2.975	0.0845	0.6091	G	0.18	C	936:186	916:220	2.977	0.0845	0.3197
rs9394697	40,529,146	GA	A	0.456	G	281	260	0.815	0.3666	0.8291	A	0.479	A	556:566	524:608	2.406	0.1208	0.4111
rs11962841	40,543,639	AG	G	0.166	A	166	140	2.209	0.1372	0.7817	G	0.135	A	986:142	974:164	1.611	0.2043	0.031
rs9296336	40,548,746	AG	G	0.213	A	195	169	1.857	0.173	0.5304	G	0.234	A	874:254	859:275	0.948	0.3303	0.5323
rs1433731	40,562,386	AC	C	0.287	A	241	220	0.957	0.328	0.8262	C	0.325	A	779:347	744:386	2.872	0.0901	0.1962
rs6925136	40,566,237	CA	A	0.159	C	160	133	2.488	0.1147	0.717	A	0.141	C	975:149	961:169	1.343	0.2465	0.0821
rs2077719	40,566,537	AG	G	0.293	A	238	232	0.077	0.782	1	G	0.295	A	811:317	776:348	2.21	0.1371	0.0955
rs9357332	40,568,204	TC	C	0.195	T	185	163	1.391	0.2383	0.8346	C	0.17	T	943:183	937:201	0.799	0.3713	0.2904
rs9369207	40,569,897	AG	G	0.158	A	153	137	0.883	0.3474	1	G	0.141	A	977:149	967:171	1.5	0.2206	0.1299
rs1114593	40,570,829	CT	T	0.095	T	105	91	1	0.3173	0.8465	T	0.078	C	1044:82	1039:95	0.938	0.3327	1
rs2504832	40,576,968	TA	A	0.431	A	288	267	0.795	0.3727	0.8852	A	0.399	T	683:445	675:457	0.2	0.655	0.2328
rs1036287	40,583,786	CT	T	0.4	T	276	252	1.091	0.2963	0.5567	T	0.365	C	719:407	719:419	0.111	0.7393	0.2518
rs9394701	40,584,431	CT	T	0.112	T	120	100	1.818	0.1775	1	T	0.077	C	1052:76	1035:99	3.145	0.0761	0.9626
rs12663856	40,585,226	AT	T	0.093	A	104	83	2.358	0.1246	0.7152	T	0.117	A	995:131	1000:134	0.018	0.8928	0.5813
rs2235705	40,594,137	CT	T	0.365	T	267	242	1.228	0.2678	0.5065	T	0.332	T	376:748	374:762	0.071	0.7893	0.9127
rs2281263	40,595,767	GA	A	0.172	A	175	157	0.976	0.3232	0.4748	A	0.106	G	1008:116	1013:123	0.154	0.695	0.7516
rs909987	40,596,619	CT	T	0.428	T	273	267	0.067	0.7963	0.5646	T	0.411	T	466:662	467:673	0.028	0.8666	0.517
rs382023	40,599,218	TC	C	0.462	T	279	257	0.903	0.342	0.4462	C	0.47	C	529:591	526:598	0.043	0.8365	0.753
rs7356878	40,602,833	AC	C	0.478	C	299	275	1.003	0.3165	0.7512	C	0.493	A	573:553	573:563	0.045	0.8313	0.8421
rs9471364	40,602,943	CG	C	0.488	C	304	281	0.904	0.3416	0.3395	G	0.475	G	539:587	536:600	0.107	0.7441	0.6489
rs9471365	40,611,372	AG	G	0.232	G	212	208	0.038	0.8453	0.4275	G	0.32	G	366:762	360:780	0.196	0.6578	0.3687
rs2196100	40,618,633	TC	C	0.163	T	166	153	0.53	0.4667	0.831	C	0.246	C	288:840	269:869	1.096	0.2951	0.8503
rs2395749	40,631,842	CT	T	0.235	T	213	208	0.059	0.8075	0.6018	T	0.353	T	408:718	391:747	0.872	0.3504	0.4635
rs10947897	40,638,778	TG	G	0.244	T	217	210	0.115	0.7348	0.7107	G	0.343	G	389:737	388:750	0.051	0.8208	0.0805
rs846520	40,672,703	GC	C	0.274	G	254	238	0.52	0.4707	0.0425	C	0.399	C	457:669	448:692	0.392	0.5314	0.6286

[a]The physical position (base) on chromosome 6 by the NCBI Build 35 map.

[b]P values for deviation from Hardy-Weinberg equilibrium.

[c] The allele count ratio (the over-represented allele : the under-represented allele)

[d]These markers were nominally significant by single-marker test. After correction for multiple-testing by 10,000 permutations, P values for rs752556, rs2033528, and rs6910503 were 0.4438, 0.4946, and 0.6658, respectively.
